# Bacteria-soil–plant linkages underlie the mosaic structure of the soil bacterial communities in near-natural stands of Białowieża Primeval Forest

**DOI:** 10.1038/s41598-026-40694-1

**Published:** 2026-03-13

**Authors:** Justyna M. Drewnowska, Wioleta Lewandowska, Piotr Zieliński, Piotr Jadwiszczak, Bogdan Jaroszewicz, Andrzej Keczyński, Olga Hummel, Piotr Majewski, Izabela Święcicka

**Affiliations:** 1 Department of Microbiology and Biotechnology, Faculty of Biology, University of Bialystok, 15-245 Bialystok, Poland; 2 Laboratory of Applied Microbiology, Faculty of Biology, University of Bialystok, 15-245 Bialystok, Poland; 3 Doctoral School, University of Bialystok, 15-245 Bialystok, Poland; 4 Department of Environmental Protection, Faculty of Biology, University of Bialystok, 15-245 Bialystok, Poland; 5 Laboratory of Paleobiology, Faculty of Biology, University of Bialystok, 15-245 Bialystok, Poland; 6 Białowieża Geobotanical Station, Faculty of Biology, University of Warsaw, 17-230 Bialowieza, Poland; 7 Białowieża National Park, 17-230 Bialowieza, Poland; 8 Reference and Bibliometric Analysis Department, University of Lodz Library, University of Lodz, 90-237 Lodz, Poland; 9 Department of Microbiological Diagnostics and Infectious Immunology, Faculty of Pharmacy With the Division of Laboratory Medicine, Medical University of Bialystok, 15-222 Bialystok, Poland

**Keywords:** Soil bacterial communities, Metataxonomy, Carbon substrate utilization, Temperate primary forests, Understorey vegetation, Soil physicochemical properties, Bacteria, Environmental microbiology, Soil microbiology, Microbial communities, Metagenomics, Microbial ecology, Microbiome, Biodiversity, Forest ecology, Microbial ecology, Microbiology, Ecology, Environmental sciences

## Abstract

Primary temperate forests serve as a natural framework for studying linkages between vegetation, soil properties and microbial communities under minimal human disturbance. Here, we characterize how soil bacterial communities and functional potential vary across five dominant forest types of the Białowieża National Park, representing a natural mosaic of vegetation and edaphic conditions. Using full-length *16S rRNA* Oxford Nanopore sequencing, functional profiling via BIOLOG EcoPlates, and applying multivariate analyses, we detected clear differences in bacterial composition and carbon-substrate utilization profile among forest types. Distance-based redundancy analysis (dbRDA) identified soil pH as the primary abiotic gradient shaping bacterial communities, while RLQ, fourth-corner and multiblock sPLS analyses consistently supported bacteria-soil-vegetation linkages. Three consistent ecological clusters emerged across the forest mosaic. Coniferous forests with acidophilic bacterial assemblages linked to strongly acidic soils and ericaceous understoreys, broadleaf forests with bacterial genera associated with moderately acidic, nutrient-depleted soils and shade-tolerant vegetation, and alder forests characterized by richer, more metabolically active microbial communities occurring in less acidic soils with tall-herb understoreys. Mixed forests displayed broad internal variability, reflecting their wide range of vegetation and soil conditions. Overall, environmental filtering structures distinct bacterial communities of this primary temperate forest, providing a valuable baseline for future plant-soil-microbiome studies.

**Supplementary Information:**

The online version contains supplementary material available at 10.1038/s41598-026-40694-1.

## Introduction

The Global Forest Resources Assessment 2020 from the Food and Agriculture Organization (FAO) indicates that forest biomes cover over four billion hectares of terrestrial surface, playing a crucial role as carbon sinks, influencing the geochemical cycles of various elements, and stabilizing the climate^1,2^. In particular, soil microorganisms in forest ecosystems play a central role in nutrient cycling and plant growth^2–5^. On the other hand, amidst ongoing global warming and environmental changes, periods of intensified microbial activity can accelerate the decomposition of carbon and nitrogen stocks in forest soils, temporarily increasing greenhouse gas emissions^4,6^. As a result of complex underground interactions, forest vegetation and soil microorganisms create a network of interdependencies, which has attracted increasing scientific interest in recent years^7–10^.

Natural forests, with their multi-layered vegetation and diverse soil chemistry, harbor a great diversity of soil microorganisms. The highest microbial biomass and metabolic activity typically occur in the topsoil, shaped by pH, organic matter input, and vegetation composition^3,10,11^. Historical disturbances (e.g., fires or bark beetle outbreaks) and ongoing processes, such as root system development, generate microhabitats with unique microclimates, further enhancing soil heterogeneity^2,12–15^. This variability influences both the taxonomic structure and the metabolic potential of soil microbial communities^10,11,16^, which additionally respond to seasonal shifts and short-time events such as rainfall, drought, snowmelt, or root activity^12,17–19^.

Among the biotic factors influencing forest soil microbiome, vegetation plays a central role. Tree species identity and diversity affect soil microbial richness and community composition^5,10,20–22^, yet plant-soil microbiomes are shaped by multiple ecological filters rather than forming a unified ‘holobiont’^7^. Although linkages between trees and soil microbial communities are relatively well described^5,10,22–27^, microbial associations with understorey vegetation, especially in natural temperate forests, remain poorly understood^28–31^. Meanwhile, understorey vegetation, as a crucial component of forest ecosystems, plays a significant role in soil processes through (i) supplying high-quality litter and root-derived carbon inputs that regulate organic matter decomposition and nutrient cycling, (ii) modifying soil microclimatic and physical conditions, including structure and moisture, and (iii) directly influencing soil microbial communities by enhancing microbial biomass, enzymatic activity, and the formation of rhizosphere ‘hotspots’ of biogeochemical activity^28–33^. In turn, soil microorganisms reciprocally regulate nutrient availability, creating feedback loops that can influence the dominance of understorey species^28^. This implies a potential link between specific understorey plant species and particular bacterial taxa, whereby plant identity and functional traits help structure microbial communities and their functional diversity in forest soils^30^. A growing number of studies provide empirical support for these species-specific linkages^28–30^. For example, *Aegopodium podagraria,* a common understorey species in riparian forests, has been reported to influence soil microbial functioning. Soils under monospecific patches of *A. podagraria* display the highest respiration rates, elevated alkaline phosphatase activity, and increased Gram-negative bacterial and fungal biomass. In contrast, plots with mixed herbaceous vegetation showed weaker or negligible effects on soil microbial parameters, illustrating that plant species identity may be more influential than plant diversity in shaping forest soil microbial communities^30^.

**Fig. 1:**
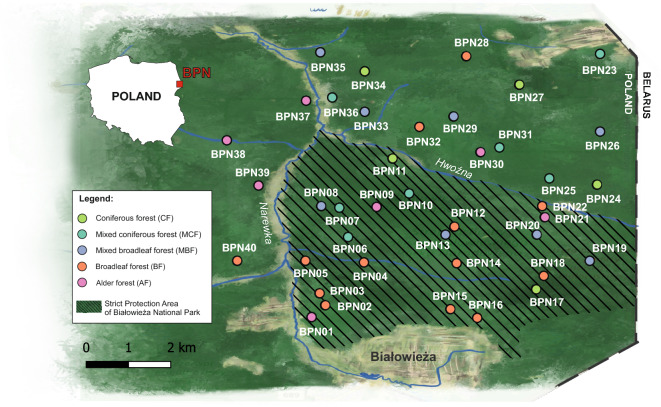
Sampling location with plot number and forest types within the Białowieża National Park. The map was generated in QGIS 3.44.7 (https://qgis.org) using OpenStreetMap base layers and subsequently refined graphically in CorelDRAW 22.1.1.523 to enhance visual clarity.

The Białowieża Primeval Forest, spanning 1500 km^2^ across Poland and Belarus, represents one of the last remaining lowland temperate forests in Europe that has retained its near-natural structure and ecological continuity. Owing to centuries of protection as a royal hunting ground since the fourteenth century, and later, strict conservation within the Białowieża National Park (established in 1921), its core areas have been largely excluded from direct human disturbance (Fig. 1). The forest’s designation as a UNESCO World Heritage site in 1979 further ensured minimal intervention, with human activity restricted to scientific monitoring and maintenance of a small number of trails^34^. This unique management history provides an exceptional opportunity to study the composition and functional potential of forest soil microbial communities, without the need to consider direct human interference.

Although the macrobiota of the Białowieża Primeval Forest, including vascular plants, invertebrates, and vertebrates, has been intensively investigated^34,35^, research on its soil microbiota remains limited^36^. Existing microbiological studies have mostly focused on selected bacterial taxa, such as members of the *Bacillus cereus* group^37–39^ or *Pseudomonas* spp.^40^, rather than on whole-community patterns. Consequently, fundamental questions about the diversity, functional potential, and ecological drivers of bacterial communities in this primary forest remain largely unanswered.

In this study, we addressed how soil bacterial communities vary across forest habitats within a primary temperate forest ecosystem and how these patterns are associated with both biotic factors (locally dominant plant species and understorey vegetation) and abiotic soil properties (including pH, moisture, organic matter content, and nutrient status). Given the limited number of studies on the soil microbiota of European primary forests, we aimed to (i) characterize variation in soil bacterial community composition and metabolic potential (assessed as carbon-substrate utilization) across five dominant forest types of the Białowieża National Park (BPN), (ii) determine how soil physicochemical properties influence microbial community structure and functional heterogeneity, and (iii) assess the extent to which vegetation composition shapes soil bacterial communities. Together, these analyses outline the diversity and functional potential of soil microbial communities in a minimally disturbed European forest, providing a baseline for research on plant-soil-microbiome linkages in temperate ecosystems.

## Results

### Variation in soil bacterial communities across five dominant forest types

To evaluate how forest type shapes soil microbial communities, we first quantified total bacterial cell numbers using flow cytometry. Bacterial abundance differed significantly among forest types (ANOVA, *p* < 0.01), with coniferous forests (CF) showing markedly higher counts than alder (AF) and broadleaf (BF) stands (Supplementary Tables S1 and S2).

We next assessed taxonomic composition and diversity of soil bacterial communities using full-length 16S *rRNA* gene sequencing. Across all samples, 47.9 million reads were obtained and classified into 1648 bacterial genera. Alpha diversity metrics differed significantly among forest types (Kruskal–Wallis, *p* < 0.01 for all indices; Supplementary Table S2), with AF exhibiting the highest richness, evenness, and overall diversity, followed by BF, mixed broadleaf (MBF), and mixed coniferous (MCF) stands, whereas CF consistently displayed the lowest values. Post hoc tests confirmed contrasts between AF and the coniferous-dominated forest types (CF, MCF), and between CF and BF (*p* < 0.05). Similar patterns were observed at higher taxonomic ranks.

**Fig. 2:**
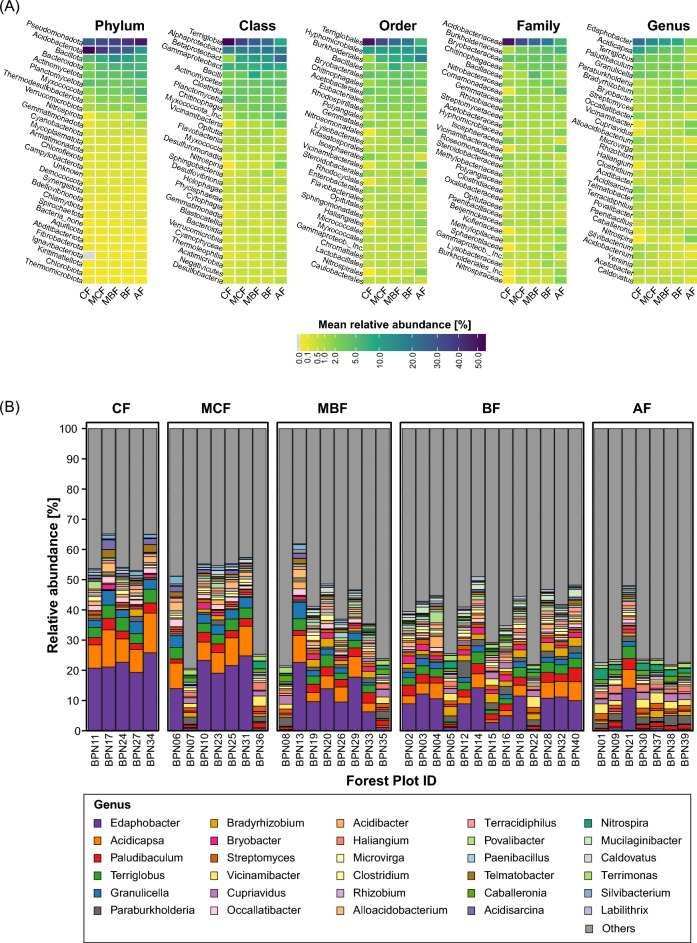
Composition and relative abundance of dominant bacterial taxa in topsoil samples from different forest types. (**A**) Heatmaps showing the mean relative abundance of the 30 most abundant bacterial taxa at different taxonomic levels (Phylum, Class, Order, Family, and Genus) in topsoil samples from coniferous (CF), mixed coniferous (MCF), mixed broadleaf (MBF), broadleaf (BF), and alder forests (AF). (**B**) Stacked bar charts showing the relative abundance of the 30 most abundant bacterial genera in individual plots from the same forest types. Only taxa representing ≥ 1% of the total abundance in at least one sample were retained; all remaining taxa were grouped as *Others* (grey).

Across forest types, soil bacterial communities were dominated by Pseudomonadota and Acidobacteriota, which together accounted for > 50% of all reads in each sample (Fig. 2A, Supplementary Fig. S1, Table S3). Their relative contribution followed contrasting forest-type gradients. Pseudomonadota increased from CF toward AF and BF forests, whereas Acidobacteriota predominated in CF stands. Within Pseudomonadota, Betaproteobacteria were particularly associated with AF soils, with high contributions of genera such as *Paraburkholderia, Cupriavidus, Caballeronia* (from Burkholderiaceae family)*, Polaromonas* (Comamonadaceae), and *Usitibacter* (Nitrosomonadales), while *Acidobacteriaceae* (e.g. *Edaphobacter, Acidicapsa, Terriglobus, Granulicella*, *Acidisarcina,* and *Alloacidobacterium*) dominated CF stands. (Fig. 2, Supplementary Fig. S1). AF soils were further characterised by increased contributions of *Vicinamibacter* (Acidobacteriota), *Streptomyces* (Actinomycetota), *Haliangium* (Myxococcota), and *Nitrospira* (Nitrospirota), whereas MBF showed relatively higher abundance of Bacillota. A full list of taxa and their relative abundances is provided in Supplementary Table S3.

**Fig. 3:**
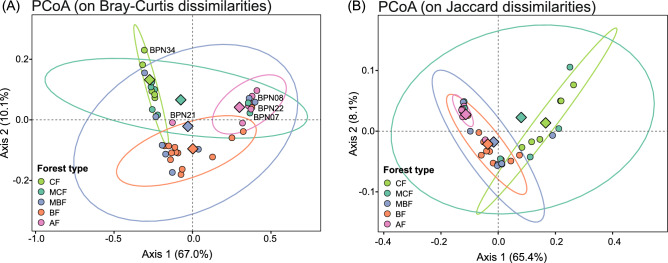
Principal coordinates analysis (PCoA) of bacterial community β-diversity at the genus level. PCoA based on (**A**) Bray–Curtis dissimilarities, and (**B**) Jaccard (presence/absence) dissimilarities, illustrates compositional differences in bacterial communities among forest types (CF, coniferous forest; MCF, mixed coniferous forest; MBF, mixed broadleaf forest; BF, broadleaf forest; AF, alder forest). Ellipses denote 95% confidence intervals for each forest type, and diamonds indicate group centroids (mean coordinates). Sample labels mark statistical outliers identified as points lying beyond the 95% confidence ellipse for their respective forest type. Percentages in axis labels denote the proportion of variance explained by each ordination axis.

Principal coordinates analysis (PCoA) based on Bray–Curtis dissimilarities was used to visualize patterns in soil bacterial community composition among forest types, with the first two axes explaining 77.1% of the total variation (67.0% and 10.1% for axes 1 and 2, respectively; Fig. 3A). The ordination showed a clear separation of AF and CF forest communities along the first axis, whereas bacterial assemblages from BF formed a distinct but partly overlapping cluster. In contrast, bacterial communities from mixed forest types (MCF and MBF) exhibited broad within-type variability. Formal statistical testing using PERMANOVA indicated significant differences in community composition among forest types (pseudo-R^2^ = 0.33, df = 4, *p* < 0.001). Pairwise PERMANOVA comparisons showed that AF bacterial communities differed significantly from all other forest types (BH-adjusted *p* < 0.05), with an additional significant difference observed between BF and CF (Supplementary Table S4). However, PERMDISP revealed variation in within-group dispersion (df = 4, *p* < 0.05), indicating that part of the observed differentiation arises from differences in within-type heterogeneity rather than differences in group centroids alone.

Ordinations based on Jaccard dissimilarities (presence/absence) complemented the Bray–Curtis results by emphasizing differences in taxon occurrence rather than relative abundance. While Jaccard-based ordinations still showed a clear separation of AF and CF bacterial communities, they revealed substantially greater overlap among other forest types (Fig. 3B). These results indicate that bacterial assemblages within different forest types share a large fraction of genera, and that habitat differences are not primarily driven by complete taxon turnover. Instead, the contrast between Bray–Curtis and Jaccard results suggests that compositional differences among forest types, particularly between AF and CF, are largely driven by shifts in the relative abundance of dominant or core taxa, whereas communities in BF and mixed forests are characterized by more heterogenous assemblages with similar taxon pools but variable dominance structure. PERMANOVA based on Jaccard dissimilarities confirmed significant differences among forest types (pseudo-R^2^ = 0.38, df = 4, *p* < 0.001), while pairwise comparisons showed that AF bacterial communities differed markedly from all other forest types, CF from BF and MBF, and BF from MCF (Supplementary Table S4). PERMDISP indicated significant differences in within-type dispersion (df = 4, *p* < 0.001), meaning that part of the Jaccard-based separation among forest types arises from differences in heterogeneity rather than shifts in taxon identity alone. Non-metric multidimensional scaling (NMDS) ordinations based on Bray–Curtis and Jaccard dissimilarities yielded patterns consistent with the PCoA results and are therefore presented in the Supplementary Fig. S2.

Ecological indicators derived from bacterial phylum-level relative abundances revealed functional differences among forest types (Supplementary Table S5). While the Bacillota/Bacteroidota (F/B) ratio indicated a consistently greater contribution of Bacillota in all forest types, both the Pseudomonadota/Acidobacteriota (P/A) ratio and the copiotroph/oligotroph index were significantly higher in BF and AF forests than in CF (BH-adjusted *p* < 0.01), indicating a shift toward more copiotrophic soil bacterial communities in broadleaf- and alder-dominated stands.

### Functional diversity of soil bacterial communities

Microbial functional potential was evaluated using Biolog® EcoPlates™, which quantifies the capacity of soil communities to utilize 31 carbon substrates. Across forest types, the overall patterns in metabolic activity mirrored the diversity structure observed in the *16S rRNA* dataset. AF microbial communities showed the highest metabolic activity as indicated by average well-color development (AWCD), followed by BF, MBF, and MCF stands, whereas CF soils displayed minimal AWCD values (Supplementary Fig. S3, Supplementary Table S6). In addition, patterns across the five functional substrate categories after 96 h incubation were consistent (Supplementary Fig. 3B).

Functional alpha-diversity indices derived from Biolog EcoPlate™ data exhibited pronounced differences among soil microbial communities from different forest types (Supplementary Table S1, Supplementary Fig. S4). Metabolic richness (number of utilized substrates), Shannon diversity, and Simpson diversity were significantly higher in AF than in CF, MCF, and MBF stands. Evenness did not differ significantly among forest types, indicating a relatively uniform distribution of substrate utilization in all microbial assemblages.

**Fig. 4:**
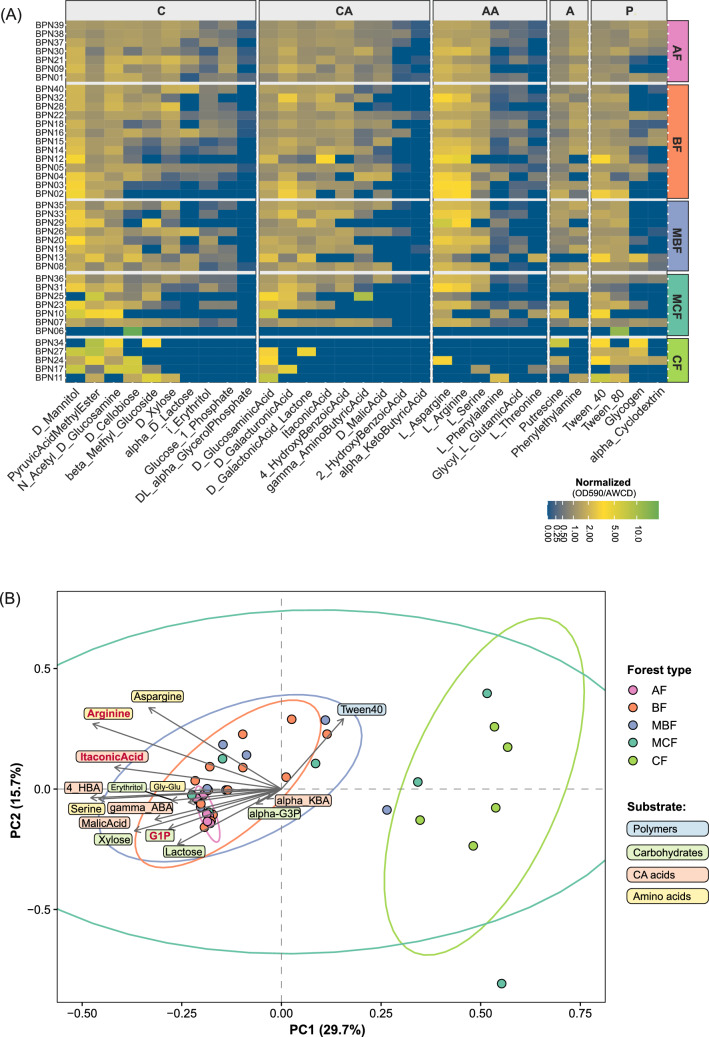
Relative substrate utilization patterns among different microbial communities from Białowieża National Park. (**A**) Heatmap showing relative carbon substrate utilization profiles in soil microbial communities based on individual substrate utilization normalized to AWCD (OD590/AWCD) after 96 h incubation. The heatmap uses a *log1p* color-scale transformation [log(1 + x)] to enhance contrast among low-activity substrates. Forest type: AF, alder forest; BF, broadleaf forest; MBF, mixed broadleaf forest; MCF, mixed coniferous forest; CF, coniferous forest. Substrate categories: P, Polymers; C, Carbohydrates; CA, Carboxylic and ketonic acids; AA, Amino acids; A, Amines/amides. (**B**) Principal Component Analysis (PCA) biplot based on Hellinger-transformed, AWCD-normalized substrate utilization profiles from Biolog EcoPlates™. The plot displays the first two principal components (PC1 and PC2), which together account for 45.4% of the total variance. Seven components were required to explain ≥ 80% of the variance. To improve interpretability, arrows represent only substrates (n = 15) showing significant differences among forest types (raw Kruskal–Wallis *p* < 0.05). Substrates that remained significant after Benjamini–Hochberg correction are highlighted in red. Ellipses denote 95% confidence regions around group centroids and are provided for visualization of within-group dispersion in each forest type.

Relative substrate utilization profiles (OD590/AWCD) also differed markedly among forest types (Fig. 4A). In CF and MCF soils, microbial communities showed the strongest relative preference for carbohydrates, followed by polymers, while amino acids consistently represented the last utilized substrate category. In contrast, the profile in AF was dominated by amines/amides, carbohydrates, and amino acids, with polymers showing the weakest relative utilization. BF communities preferentially utilized amino acids and amines/amides, while polymers remained the last responsive category. MBF samples showed the highest relative utilization of amino acids and carbohydrates. Taken together, these results indicate clear differences among microbial communities in carbon-use strategies.

The Principal Components Analysis (PCA) based on Hellinger-transformed, AWCD-normalized substrate-use profiles revealed distinct metabolic fingerprints across forest types (Fig. 4B). AF, BF and MBF soil communities, characterized by higher substrate utilization, clustered together on the negative side of PC1 axis (29.7% of the explained variance), whereas CF, showing reduced metabolic response, formed a separate cluster on the positive side. The broad confidence region of MCF reflects its high within-type heterogeneity. Substrates contributing most strongly to the separation of forest types included itaconic acid, glucose-1-phosphate, and L-arginine (BH-significant substrates, *p* < 0.05).

Spearman correlations between the relative abundance of dominant bacterial genera and relative substrate utilization profiles revealed consistent taxon-function associations across forest types (Supplementary Figs. S5 and S6). In all forest types (except CF), several genera, including *Terrimonas, Cupriavidus, Haliangium, Caballeronia, Vicinamibacter,* and *Nitrospira,* showed consistently positive correlations with a broad range of carbon substrates. These taxa were repeatedly associated with samples exhibiting higher levels of substrate utilization, indicating that they represent characteristic components of microbial communities with elevated metabolic potential in the studied soils.

### Environmental drivers of soil bacterial communities

To identify key environmental drivers of bacterial community structure, we quantified key topsoil physicochemical parameters (Supplementary Tables S1, and S7). Among all variables, soil pH showed the strongest and most consistent differentiation among forest types. AF soils were significantly less acidic (pH 5.24) than CF (pH 2.55) and MCF (pH 3.66), while BF (pH 3.95) also differed markedly from CF (BH corrected *p* < 0.05). In contrast, most other physicochemical properties did not distinguish AF from CF, despite their pronounced divergence in bacterial community composition, with significant differences in SOM, SMC, SOC, TN, C:P ratio, and ash content occurring mainly between AF and BF.

Correlation analysis among all soil properties (Supplementary Table S8) revealed that SOM, SOC, SMC, TN, TP, C:P ratio formed a tightly linked group (|ρ|> 0.7) inversely related to ash content. Because these variables represent the same underlying environmental gradient, they were reduced to one predictor (SMC) to avoid redundancy and multicollinearity in downstream analyses.

**Fig. 5:**
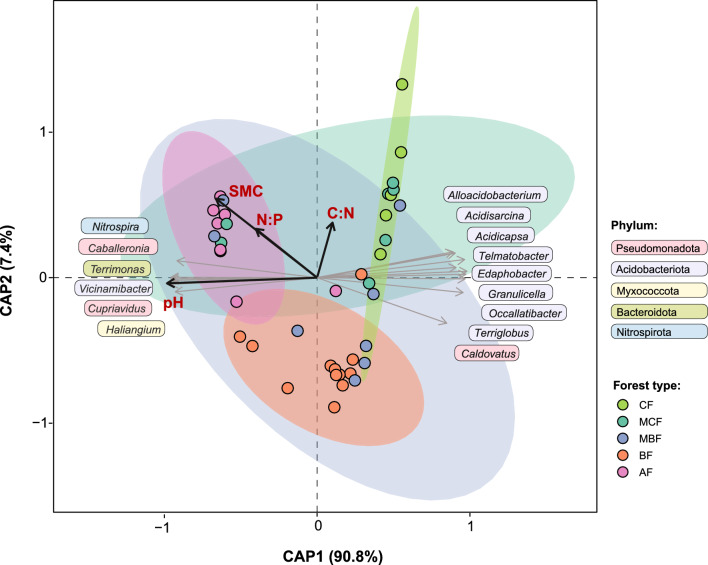
Distance-based redundancy analysis (dbRDA) showing** r**elationships among forest types, environmental variables, and bacterial community composition at the genus level. Ellipses represent 95% confidence intervals for each forest type (AF, alder forest; BF, broadleaved forest; MBF, mixed broadleaved forest; MCF, mixed coniferous forest; CF, coniferous forest), Black vectors indicate significant environmental predictors (pH; SMC, soil moisture content; C:N ratio; and N:P ratio), and grey vectors represent genera significantly fitted to the ordination using *envfit* (*p* < 0.05). Arrow lengths reflect the strength of each variable’s correlation with the constrained ordination axes.

Using this reduced variable set, distance-based redundancy analysis (dbRDA) indicated that soil properties collectively explained a substantial proportion of the variation in bacterial community composition (*p* = 0.001; adjusted R^2^ = 0.645). The first constrained axis (CAP1) captured 90.8% of the explained variation, whereas CAP2 accounted for an additional 7.4% (both statistically significant; *p* = 0.001) (Fig. 5).

The ordination biplot revealed two main environmental gradients that were associated with variation in bacterial community structure. CAP1 was primarily related to soil pH, with additional contributions from SMC and N:P. CAP2 reflected a weaker gradient associated with SMC as well as stoichiometric ratios (C:N and N:P). Since SMC represents a strongly correlated set of variables (SOM, SOC, TN, TP, C:P, and ash content), it effectively summarizes the major multivariate edaphic gradient observed across forest types.

Consistent with these gradients, AF communities occupied the high-pH end of CAP1, and were also located in the region associated with higher SMC and elevated N:P and C:N ratios. CF communities clustered toward the opposite, low-pH end of CAP1, while similarly aligning with the higher-SMC and higher-ratio region in the CAP1/CAP2 space. BF communities occupied intermediate positions along CAP1, extending across both its negative and positive sides, while remaining associated with lower SMC and stoichiometric ratios along CAP2. MCF and MBF exhibited the greatest within-type dispersion, with broad confidence ellipses encompassing much of the ordination space and overlapping other forest types.

Fitted genus vectors (*envfit*) supported these associations. Genera enriched in AF soils (e.g., *Terrimonas, Cupriavidus, Vicinamibacter, Haliangium, Caballeronia, Nitrospira*) aligned with higher pH, whereas acidophilic genera, such as *Edaphobacter, Acidicapsa, Terriglobus,* and *Granulicella,* were associated to the low-pH conditions characteristic of CF soils.

### Vegetation structure and its relationship to soil bacterial communities

To provide environmental context for soil microbial variation, we characterized vegetation structure and composition at the soil sampling location across forest types (Supplementary Table S9). Vegetation cover and vertical structure differed among forest types, with significant differences detected primarily in the canopy and ground layers (Supplementary Fig. S7).

Across all plots, a total of 188 vascular plant species were recorded, and species richness varied markedly among forest stands (Supplementary Fig. S8). Coniferous forests (CF) exhibited the lowest plant richness, mixed forest (MCF and MBF) showed intermediate values, and broadleaf (BF) and alder forests (AF) were characterized by substantially higher richness. These patterns indicate strong differences in vegetation structure and diversity among forest types, providing an important ecological background for interpreting soil microbial-vegetation associations.

To address our research question concerning the role of vegetation composition as a biotic driver of soil bacterial communities, we examined patterns of understorey species composition across forest types. Understorey vegetation differed significantly among forest types (PERMANOVA, R^2^ = 0.38, df = 4, *p* = 0.001), reflecting a clear forest-type gradient. The first two PCoA axes explained 27.4% and 14.3% of variance, respectively (Supplementary Fig. S8). CF formed a compact cluster characterized by low-diversity, ericaceous-dominated understorey, whereas AF occupied the opposite region of ordination space, reflecting tall-herb understorey (e.g., *Urtica dioica*) accompanied by *Carex remota*. BF clustered apart from both CF and AF, reflecting their distinct herb-layer communities dominated by *Oxalis acetosella* and other shade-tolerant taxa. MCF and MBF overlapped broadly, indicating transitional understorey assemblages. Moreover, these differences were not driven by unequal within-group dispersion (betadisper, *p* = 0.13). Pairwise PERMANOVA confirmed the strong dissimilarity between forest types (*p* < 0.01), except MCF vs. MBF, aligning with their overlap in ordination space. Overall, understorey vegetation exhibits strong forest-type specificity, with CF and AF representing the most contrasting communities, providing a key biotic context for interpreting soil bacterial community patterns and their integration with vegetation in subsequent multivariate analyses.

To assess the extent to which understorey vegetation structure explains variation in soil bacterial community composition, we first used a dbRDA constrained by the first two ordination axes of the vegetation (VegPCoA1-2). Together, these vegetation gradients accounted for 41.7% of the variation in understorey composition (Supplementary Fig. S8). The dbRDA model showed that understorey vegetation composition explained a substantial fraction of the variation in bacterial communities (adjusted R^2^ = 0.564; constrained variance = 58.7%) (Supplementary Fig. S9). The overall model was highly significant (*p* = 0.001), and both constrained axes contributed significantly to explaining variation (*p* = 0.001). CAP1 captured 89.9% of the constrained variance, with CAP2 explaining the remaining 10.1%. Vector fitting confirmed that both VegPCoA1 and VegPCoA2 were strongly associated with the dbRDA ordination (both *p* = 0.001), indicating that the two main vegetation gradients jointly structure bacterial community turnover. The ordination plot revealed clear differentiation among CF vs. AF, as well as CF vs. BF along these vegetation-driven gradients, while MBF and MCF showed broader distribution, consistent with intermediate understorey composition patterns.

The fourth-corner analysis detected numerous significant associations between understorey species and bacterial genera (FDR-adjusted *p* < 0.05), resulting in 62 significant plant-bacteria links (Supplementary Fig. S10). *Nitrospira* and *Edaphobacter* showed the most consistent and contrasting patterns. For example, *Nitrospira* displayed higher positive correlations with *Impatiens noli-tangere, Geranium robertianum, U. dioica, Lythrum salicaria, Filipendula ulmaria, Myostis palustris, Cirsium oleraceum,* and *Lycopus europaeus*, and negative with *Calamagrostis arundinacea.* In turn, *Edaphobacter* showed positive linkeages with *Vaccinium myrtillus, V. uliginosum, C. arundinacea, Trientalis europaea,* and *Molinia caerulea,* and negative with *U. dioica, L. salicaria, F. ulmaria, M. palustris,* and *L. europaeus.* These opposite correlation directions reflect the distinct ecological niches of both genera and mirror major vegetation gradients across forest types.

**Fig. 6:**
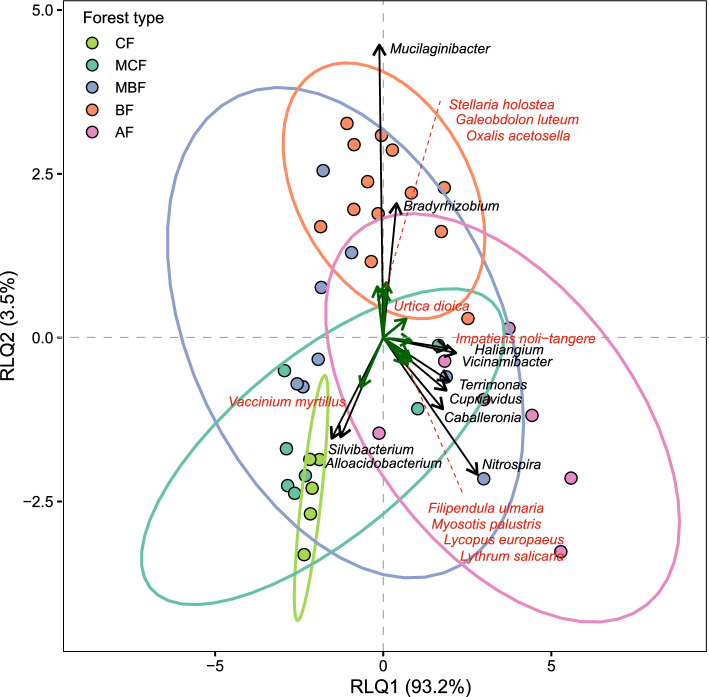
RLQ ordination summarizing the joint structure between soil bacterial communities and understorey vegetation from different forest types. RLQ biplot showing the co-structure between understorey plant composition and bacterial community composition. Points represent sampling plots with 95% confidence ellipses indicating within-type dispersion. Arrows denote the strongest bacterial genera (black) and understorey plant species (green) associated with the first two RLQ axes.

A global RLQ permutation test revealed a strong co-structure between understorey vegetation and bacterial community composition (Model 2: Obs = 0.964, *p* = 0.001). The first RLQ axis accounted for 93.2% of the co-interia, and RLQ2 for an additional 3.5% together capturing 96.7% of the total co-variation represented on the first two axes (Fig. 6). CF formed a compact cluster that was clearly separated from AF and BF along RLQ1 and RLQ2, respectively. Mixed forests showed broader dispersion, reflecting their more heterogeneous and intermediate character. Vectors associated with the CF region included *Silvibacterium, Alloacidobacterium,* and *V. myrtillus.* In contrast, the AF region aligned strongly with *Nitrospira, Caballeronia, Cupriavidus, Terrimonas, Haliangium, Vicinamibacter,* as well as understorey species such as *I. noli-tangere, F. ulmaria, M. palustris, L. europaeus,* and *L. salicaria.* The BF region was associated mainly with *Bradyrhizobium, Mucilaginibacter,* and plant species such as *Stellaria holostea, Galeobdolon luteum,* and *O. acetosella.*

### Joint plant-soil-microbiome structure revealed by block sPLS

To integrate the major ecological datasets and identify key cross-domain linkages among soil properties, understory vegetation, and bacterial communities, we employed a multiblock sparse partial least squares (block-sPLS) analysis. The final block-sPLS model (30 bacterial genera × 30 plant species, six soil variables) captured a strong shared structure among bacteria, understorey vegetation and soil. Correlations between block variates were high, with mean absolute correlations of 0.84 between Bacteria and Plants, 0.71 between Plants and Soil, and 0.63 between Bacteria and Soil (overall mean |cor|= 0.725; *p* = 0.001).

**Fig. 7:**
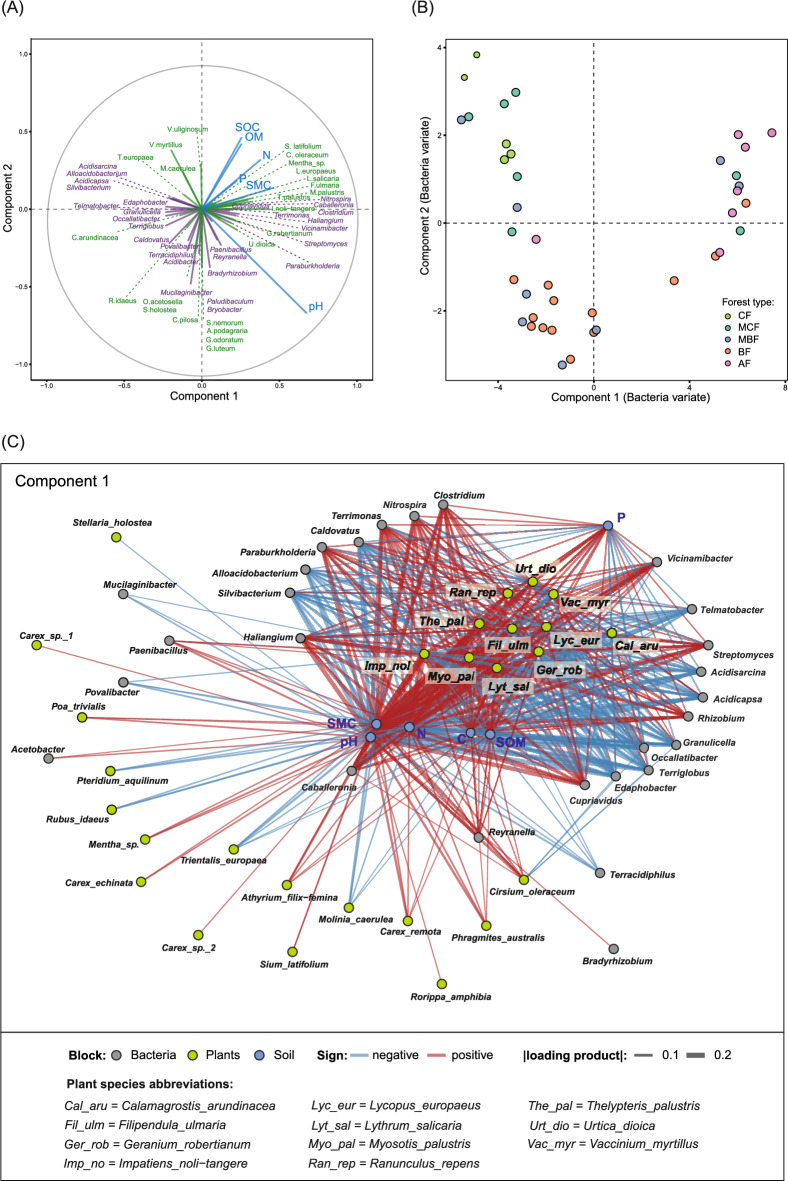
Multiblock integration of bacterial communities, understorey vegetation, and soil properties using sparse partial least squares (sPLS). (**A**) Correlation circle plot showing loadings of bacterial genera (violet), understorey plant species (green), and soil variables (blue) (pH; SMC, soil moisture content; SOC, soil organic carbon; OM, organic matter; N, nitrogen; P, phosphorus) on the first two block.sPLS components. Vectors are scaled to fit within the unit circle, and low loadings were omitted. (**B**) Sample scores for the bacterial block on components 1 and 2, with points representing plots from different forest types (CF, coniferous forest; MCF, mixed coniferous forest; MBF, mixed broadleaf forest; BF, broadleaf forest; AF, alder forest). (**C**) Network representation of sPLS component 1. Nodes correspond to bacterial genera, understorey plant species and soil variables. Edges connect pairs of variables from different blocks whose products of loadings on component 1 exceed the selection threshold (≥ 0.03), with colour indicating the sign (positive or negative) and edge width proportional to |loading product|.

The first sPLS component captured the dominant multiblock gradient (Fig. 7A,B). Sample scores based on the bacterial variate showed clear separation among forest types. CF stands clustered at one end of the gradient, AF at the opposite end, BF occupied intermediate positions, while mixed forests spread along the component 1 axis. Loadings on component 1 highlighted the variables contributing most strongly to the observed ecological gradient. Several bacterial genera (e.g., *Caballeronia, Cupriavidus, Nitrospira, Terrimonas, Haliangium, Vicinamibacter*) and understorey plant species (e.g., *I. noli-tangere, L. salicaria, F. ulmaria, L. europaeus, M. palustris, U. dioica*) exhibited high positive loadings, together with soil variables pH, SMC, SOC, SOM, total N and total P (Fig. 7). In contrast, several genera belonging to Acidobacteriaceae (*Edaphobacter, Alloacidobacterium, Silvibacterium, Acidisarcina, Acidicapsa, Telmatobacter)* and conifer-associated understorey vegetation (e.g., *V. myrtillus, M. caerulea. T. europaea)* loaded strongly in the negative direction. The network representation (Fig. 7C) provides a complementary summary of these relationships by visualizing the strongest cross-block associations.

The sPLS Component 2 represented a secondary ecological gradient, with soil loadings contrasting higher pH against lower soil carbon and organic matter. Along this gradient, BF and several MBF plots clustered on one side, characterized by genera such as *Bradyrhizobium, Bryobacter, Mucilaginibacter, Paludibaculum,* and plant species including *Aegopodium podagraria, G. luteum, Galium odoratum, S. nemorum, S. holostea* and *O. acetosella.* In contrast, all other forest types, together with their associated vegetation and bacterial communities, were positioned on the opposite side of the gradient. Together, the two components identified by sPLS summarized the principal axes of shared structure across microbial, vegetation, and soil datasets.

## Discussion

The Białowieża National Park (BPN) contains a naturally assembled mosaic of forest patches, including coniferous (CF), mixed coniferous (MCF), mixed broadleaf (MBF), broadleaf (BF), and alder (AF) forests, that has persisted for centuries under minimal anthropogenic disturbance^41^. Such long-term structural heterogeneity is considered as a feature of the self-regulated and healthy forest ecosystems^42^. In this study, we investigated how variation in vegetation and soil physicochemical properties across these dominant forest types shapes the composition and functional capacity of soil microbial communities. Our results show that soil bacterial microbiota closely reflect this ecological mosaic, displaying clear differences between forest types and consistent alignment with major vegetation-soil gradients.

Soil physicochemical properties, particularly pH, explained a large proportion of the variation in bacterial communities, which is consistent with findings from numerous local and global studies on similar ecosystems^16,27,43–47^. Topsoil pH ranged from 2.55 ± 0.39 in CF, followed by 3.95 ± 0.74 in BF, and 5.24 ± 0.85 in AF. In contrast, mixed forests exhibited substantial variability in soil parameters, a pattern mirrored by their high within-type dispersion in microbial community structure. Across forest types, the soil pH gradient emerged as the primary abiotic factor associated with the formation of three taxonomically and metabolically distinct bacterial communities.

Interestingly, while BF differed markedly from both CF and AF in several other soil properties (lower SMC, SOM, SOC, total N, total P, and higher ash content), none of these variables differed significantly between CF and AF, despite the strong divergence of their bacterial communities. However, comparable total nutrient pools do not imply similar nutrient availability^48^. The strong influence of pH on bacterial community composition likely reflects a combination of direct physiological constraints and indirect effects mediated through nutrient availability and soil chemistry. Soil pH directly affects microbial cell membrane stability, enzyme activity, and energy expenditure for pH homeostasis, thereby acting as a strong environmental filter on bacterial taxa. In addition, pH exerts profound indirect effects by regulating nutrient solubility and turnover. In highly acidic soils, phosphorus becomes immobilized through binding to Fe/Al oxides^49^, whereas nitrogen accumulates in recalcitrant organic complexes, since low pH strongly suppresses extracellular enzyme activity and slows mineralization rates^50^. These conditions limit nutrient bioavailability and favor slow-growing oligotrophs, particularly *Acidobacteriaceae*^51,52^, which dominated CF soils in our study (up to 61%)*.* However, the most surprising pattern was that CF soils, despite being dominated by oligotrophs and having the lowest metabolic activity, exhibited the highest total bacterial cell counts. We hypothesize that *Acidobacteriaceae* may form large, slow-growing populations in acidic soils, where overall microbial activity is reduced, and both competition and predation pressure are likely diminished^53^. Under such conditions, acid-tolerant oligotrophic taxa can persist and even diversify despite low nutrient availability^52^.

In contrast, the less acidic AF soils supported a more heterogeneous bacterial community, with higher relative abundances of Burkholderiaceae (e.g., *Caballeronia, Cupriavidus*)*,* Vicinamibacteriaceae (*Vicinamibacter*)*,* Nitrospiraceae (*Nitrospira*), as well as Polyangiaceae (*Haliangium*)*.* AF bacterial communities also exhibited the highest metabolic potential and alpha-diversity, which aligns with their higher soil pH and other physicochemical properties. The optimal pH for plant growth and the majority of soil microorganisms is generally considered to be 5.5–6.5, a range in which plants produce large quantities of root exudates that fuel microbial growth^54^. Norman and Barrett further demonstrated that higher soil pH enhances ammonia availability and increases the richness of ammonia-oxidizing bacteria (AOB)^47^. In line with these findings, AF samples with pH > 5 showed increased relative abundance of *Nitrospira*, a genus that includes both nitrite oxidizers and comammox lineages capable to complete ammonia oxidation^55^. Their presence corresponds to the strong utilization of amines/amides and amino acids observed in AF soils. In addition, when substrate-taxon associations were examined within AF, the pattern became more heterogenous, and a broader set of bacterial genera showed significant but individually weaker correlations with particular substrate types. Such metabolic diversity likely reflects the varied taxonomic compositions of AF soil microbial communities and their gene functional potential^18^. Our study found the highest metabolic potential and diversity of bacterial communities in AF, with plant species richness also being higher in this habitat compared to other forest types. This finding is consistent with previous research, which has shown a positive relationship between plant species richness and microbial diversity in soils^28,29,56^.

Comparable to soil chemistry, understorey vegetation explained a substantial proportion of observed bacterial variation, confirming other studies showing that this vegetation layer is tightly coupled to microbial community structure^28,29^. Tall-herb understoreys (*U. dioica, I. noli-tangere, F. ulmaria, M. palustris, L. europaeus, L. salicaria*) characteristic of AF are linked positively with *Nitrospira, Haliangium, Vicinamibacter, Terrimonas, Cupriavidus,* and *Caballeronia.* Conversely, ericaceous and graminoid understoreys in CF aligned with acidophilic *Acidobacteriaceae,* while shade-tolerant understoreys (e.g., *S. holostea, G. luteum, O. acetosella*) in BF aligned with bacterial genera including *Bradyrhizobium, Bryobacter,* and *Mucilaginibacter.*

These consistent patterns across dbRDA, fourth-corner, RLQ and sPLS analyses, combined with the high β-dispersion observed in mixed forests (MBF and MCF), reveal that the studied soil bacterial communities do not form a single gradient but instead organize into three robust ecological clusters, each supported by specific vegetation, soil properties, and microbial signatures. The AF cluster is characterized by higher pH, tall-herb understoreys and metabolically versatile bacterial taxa; the CF cluster is defined by higher acidic soils, ericaceous vegetation and acidophilic oligotrophs; while BF cluster represents an intermediate but distinct assemblage associated with intermediate acidic, relatively dry, and carbon- and nutrient-depleted soils, shade-tolerant understorey plants, and bacterial genera adapted to acidic, organic-poor forest conditions^3^. The clear delimitation of AF, BF, and CF contrasts sharply with the broad internal heterogeneity of MCF and MBF, where highly variable vegetation structure and soil conditions lead to the formation of overlapping bacterial communities. The community patterns observed across forest types align with ecological theory on microbial community assembly, which emphasizes the role of deterministic processes, primarily environmental filtering, in generating distinct communities under contrasting abiotic and biotic conditions^21,57^. In CF, AF, and BF forests, relatively uniform combinations of soil pH and understorey composition produced coherent and well-separated bacterial assemblages despite spatial separation of plots. In contrast, mixed forests (MCF and MBF) showed the highest within-type taxonomic dispersion, consistent with community differentiation along multiple, spatially variable environmental gradients. Their broad spread in ordination space also matches expectations that increasing habitat heterogeneity can enhance the influence of stochastic processes^58,59^.

In conclusion, soil bacterial communities in the Białowieża National Park form three clear ecological clusters within alder, broadleaf and coniferous forests, each shaped by characteristic combinations of soil acidity and understorey vegetation. Mixed forests show high internal heterogeneity, yet the dominant forest types exhibit strong and consistent vegetation-soil-microbiome linkages across the landscape. Alder forests harbor the highest bacterial diversity and metabolic potential, coniferous forests support acidophilic oligotrophic assemblages, and broadleaf forest bacterial communities adapted to moderately acidic, nutrient-poor soils. Together, these patterns highlight the central role of deterministic environmental filtering, primarily soil pH and vegetation identity, in structuring microbial communities in this primary temperate forest, with mixed stands shaped by multiple, spatially variable filters. The emergence of such well-distinct microbial assemblages underscores the value of the Białowieża National Park as a natural laboratory for studying microbial evolution, plant–microbe feedbacks, and belowground ecological processes, as well as provides a unique baseline for future investigations of long-term temporal dynamics and seasonal shifts in bacterial communities.

## Methods

### Study area, soil sampling, and plant community structure

The study area (the Białowieża National Park) is located at an altitude of 150–160 m a.s.l (https://bpn.com.pl). According to the Köppen-Geiger climate classification, the Białowieża Primeval Forest has a humid continental climate with a warm summer (Dfb)^38^. The average annual precipitation is 625 mm, and the average annual temperature from 1985 to 2015 was 7.3 °C (the average air temperature of 16.3 °C between May and July)^60^.

Soil samples (approximately 50 g each) were collected in triplicate from a depth of 5–15 cm at 40 sites (Fig. 1) in May 2019 (n = 120). The samples were collected using a sterile corer and placed in Falcon tubes. Prior to sampling, the litter layer was removed using a sterile metal frame (15 × 15 cm). All tools were sterilized in a 10% hypochlorite solution between samplings. The soil samples were stored in a refrigerator during transport to the laboratory.

The vegetation surveys were carried out in July 2019 on 40 plots (10 m × 10 m) centered around the soil sampling point. Plant species cover was estimated using Londo’s decimal scale^61^. Three forest layers were defined: (i) understorey (all herbaceous plants, shrubs, and trees < 0.5 m high), (ii) shrub layer (shrubs and trees 0.5–6 m high), and (iii) canopy layer (trees over 6 m high). Based on the habitat map of the Białowieża National Park, the study plots were selected from (i) coniferous forests (CF) (n = 5), (ii) mixed coniferous forests (MCF) (n = 7), (iii) mixed broadleaved forest (MBF) (n = 8), (iv) broadleaved forest (BF) (n = 13), and (v) alder forest (AF) (n = 7) (Fig. 1, Supplementary Table S9). Additionally, a vegetation inventory was performed around each soil sampling point within a 50 cm diameter circle.

Total plant species richness (R) per plot was calculated as the number of vascular plant species with non-zero cover in three layers combined, reflecting overall plot-level diversity. To characterize vegetation structure, dominance was evaluated separately for each forest type and vegetation layer. Plant species fulfilling ≥ 5% constancy and ≥ 20% mean cover were classified as dominant.

Differences in plant community composition among forest types were further examined using principal coordinates analysis (PCoA) based on Bray–Curtis dissimilarities. For this analysis, only the understorey layer was used. Londo values were converted to percentage cover and square-root transformed before distance calculation to reduce the influence of highly dominant species. Dissimilarity matrices were computed using the function *vegdist* from the *vegan* package^62^*,* and PCoA ordinations were generated using the base R (version 4.3.1) function *cmdscale*^63^*.* Differences in community composition among forest types were tested using PERMANOVA (*adonis2,* 999 permutations), and homogeneity of multivariate dispersion was evaluated using *betadisper* followed by permutation tests. Pairwise PERMANOVA comparisons between forest types were conducted on the same matrix with BH-corrected *p-*values, and the ordination was visualized as a biplot.

### Soil physicochemical analyses

For physicochemical profile analysis, equal wet weights of soil from the same study plot were pooled (10 g in total). The gravimetric method was used to determine the soil moisture content (SMC) by calculating the mass of water in a soil sample as the difference between the mass of the soil sample before and after oven overnight drying at 105 °C (Memmert SF110, Germany). Dried soil samples were then ground and sieved (1 mm). For soil organic matter (SOM) determination, samples were oven-dried at 105 °C, cooled in a desiccator, and weighed before combustion at 550 °C in a muffle furnace (SNOL 3/1100, Lithuania). After combustion, the samples were cooled in a desiccator and weighed again. An estimation of SOM percentage from the loss on ignition method was calculated by the following equation:

$${\mathrm{SOM}} = \left[ {\left( {{\mathrm{IW}} - {\mathrm{FW}}} \right)/{\mathrm{FW}}} \right] \times {1}00$$

where *IW* is initial weight (oven-dry soil weight), and *FW* is final weight (soil weight after combustion).

Two types of pH were measured using a Hach-Lange pH meter (Hach HQ411d) in a 1:2.5 soil water mixture and in a 1:2 soil KCl solution (1 M) mixture^64^. Since pH_KCl_ better reflects exchangeable acidity in strongly acidic forest soils^65^, it was used in all subsequent statistical analyses, while both measurements are reported in Supplementary Table S1. Approximately 25–50 mg of dried and sieved (1 mm) soil was used for total soil organic carbon (SOC) analysis by dry combustion at 900 °C using the total organic carbon analyzer (TOC-A with SSM 5000A module (Shimadzu, Kyoto, Japan).

Total nitrogen (TN) content was measured using a Spectroquant nitrogen cell test following Koroleff’s method^66^. Soil samples were treated with an oxidizing agent and mineralized at 100 °C, then acidified with sulfuric and phosphoric acids. TN was measured spectrophotometrically with 2,6-dimethylphenol (DMP) (Spectroquant, Pharo 300, Merck, EU). Total phosphorus (TP) content was measured spectrophotometrically by perchloric acid digestion followed by the molybdate-ascorbic acid method^67^ using SpectraMax M2 (Molecular Devices Corp., Menlo Park, USA).

### Metabolic fingerprinting of soil microorganisms

Within 48 h after collection, soil suspensions (1:10) were prepared from 10 g of soil with 0.85% sterile physiological saline solution and shaken (15 min, 200 rpm, 25 °C) with 1 min vortexing (at maximum speed) every 5 min. The samples were then centrifuged for 5 min at 130 × *g,* and the supernatants were filtered through sterile cell strainers with a mesh size of 40 μm. Immediately after extraction, the suspensions were ten-fold diluted. Finally, 150 μl of 10^–2^ dilutions was used to inoculate each well in the EcoPlates™ (Biolog Inc., Hayward, CA, USA) which contained 31 different carbon sources (plus a control well) in triplicate. The microorganisms in the wells were incubated at 25 °C in the dark. The substrate utilization rate was indicated by the reduction of the tetrazolium dye, which changed from colorless to purple. Absorbance at 590 nm was measured at 24-h intervals over a period of four days using a SpectraMax M2 Multimode Plate Reader (Molecular Devices Corp.).

Before normalization and average well-color development (AWCD) calculation, all well absorbance values corrected for the control well (*Ci-R*) ≤ 0.25 were set to zero to minimize background interference and retain only metabolically meaningful substrate responses. The dynamics of substrate utilization in microbial communities were measured by the AWCD^68^, calculated using the following formula:

$$AWCD = \sum {(C_{i} - R)} /n$$

where *C*_*i*_ is the absorbance value of *i* well at 590 nm, R is the absorbance value of the control well, and *n* is the number of wells.

The calculation of metabolic functional diversity was done from one timing (96 h) using the following indices:

1. Shannon–Wiener diversity index (H’).
  $${\mathrm{H}}^{\prime } = \sum p_{i} \times \left( {{\mathrm{ln}}p_{i} } \right)$$
  Where *p*_*i*_ is the absorbance value in *i* well (C_*i*_) divided by the sum of all the *C*_*i*_ values across all utilized substrates.
2. Richness (S)—the total number of utilized substrates from 31 carbon sources calculated using an OD_590_ = 0.250 value as a threshold for a positive response.
3. Shannon evenness index (E).
  $${\mathrm{E}} = {\mathrm{H}}^{\prime } /({\text{ln S}}).$$
4. Simpson’s diversity index (D).
  $$D = 1 - \sum {p_{i}^{2} }$$

To evaluate the relative substrate utilization profiles of microbial communities, the following normalization formula was applied for each substrate:

$$R_{si} = ({C}_{i}-R)/AWCD$$

Differences in average substrate-group utilization within each forest type were tested using the Kruskal–Wallis test followed by Dunn’s post-hoc comparisons with Benjamini–Hochberg correction. For each individual substrate, differences among forest types were assessed with separate Kruskal–Wallis tests, and *p-*values were adjusted using the BH procedure to identify substrates showing significant variation in utilization.

To explore multivariate patterns of metabolic activity, we applied Principal Component Analysis (PCA) based on *R*_*si*_ values after Hellinger transformation. The analysis was performed using the ‘*prcomp’* function in the R environment^63^. Eigenvalues and the proportion of variance explained by each principal component were extracted. For visualization in the PCA biplot, we selected the 15 substrates that showed raw Kruskal–Wallis *p* < 0.05.

### Flow cytometric assessment of bacterial abundance in the soil samples

In total 1 ml of the soil supernatant with a 10^–3^ dilution from the previous step (*Metabolic fingerprinting of soil microorganisms*) was stained with 10 μl of a stock solution of SYBR® Green I (50 × in anhydrous dimethylsulfoxide, DMSO). The samples were then incubated for 15 min in the dark at room temperature and analyzed on the BD Accuri™ C6 Plus Flow Cytometer (BD Biosciences, San Jose, USA). Green and red fluorescence were measured at 600 nm (FL1 channel) and 650 nm (FL3 channel). Data were collected as FL1/FL3 dot plots with FSC-H threshold set to 1,000 to exclude small particles. Several positive controls (*Bacillus cereus* strain ATCC 10,987, *Staphylococcus aureus* strain ATCC 33,591, *Escherichia coli* strain ATCC 11,229) and water (used as a negative control) were included for optimal differentiation between stained intact microbial cells and sample background and/or instrument noise.

### Soil DNA extraction, *16S rRNA* sequencing, and bioinformatics pipeline

Total DNA was extracted from 120 soil samples in triplicates using the NucleoSpin Soil (MACHEREY–NAGEL GmbH & Co. KG, Düren, Germany) according to the manufacturer’s protocol. Briefly, 400 mg of fresh soil was transferred to an MN Bead Tube Type A, to which 700 μl of Buffer SL1 was added, and the mixture was vortexed at maximum speed for 5 min at room temperature (RT). Contaminants were precipitated by centrifugation at 11,000 × g for 2 min. The clear supernatant was transferred to a new collection tube and mixed with 150 μl Buffer SL3 by vortexing for 5 s. Afterwards, the sample was incubated at 4 °C for 5 min and centrifuged at 11,000 × g for 1 min. Next, 700 μl of the clear supernatant was loaded onto the filter of the NucleoSpin Inhibitor Removal Column and centrifuged at 11,000 × g for 1 min. The binding conditions were adjusted by adding 250 μl of Buffer SB to the supernatant. Subsequently, DNA from 550 μl sample volume was bound to the NucleoSpin Soil Column by twice centrifugation steps at 11,000 × g for 1 min. The silica membrane was washed and dried according to the manufacturer’s instruction. Finally, DNA was eluted with 50 μl of Buffer SE. DNA quality was assessed using a NanoDrop 2000 Spectrophotometer (ThermoFisher Scientific, Wilmington, USA) and agarose gel electrophoresis. DNA concentrations were determined using a Qubit 2.0 Fluorometer with a Qubit DNA HS Assay Kit (Thermo Fisher Scientific).

Total DNA was isolated in triplicates, mixed in equal amounts (20 ng per sample, n = 120), and then used for bacterial community profiling using Oxford Nanopore technology (ONT) (Oxford Nanopore Technologies plc, Oxford, United Kingdom). The library was prepared according to the ONT protocol ‘16S Barcoding Kit 1–24 (SQK-16S024)’ (16S_9086_v1_revH_14Aug2019) with recommended third-party consumables. Specific primers 27F (5’-AGAGTTTGATCMTGGCTCAG-3’) and 1492R (5’-TACGGYTACCTTGTTACGACTT-3’), containing 5’ tags that facilitate the ligase-free attachment of Rapid Sequencing Adapters, were used to amplify the entire ~ 1500 bp *16S rRNA* gene by polymerase chain reaction (PCR). Briefly, PCR amplifications were performed in three independent 50 µl reactions per sample, containing 25 µl LongAmp Hot Start 2X Master Mix (NEB, M0533S), 10 ng input DNA (previously adjusted with nuclease-free water to 1 ng/µl), 5 µl nuclease-free water and 10 µl of a unique 16S barcode. The cycling conditions were as follows: 95 °C for 1 min, 25 cycles of 95 °C for 20 s, 55 °C for 30 s, and 65 °C for 2 min, followed by a final extension at 65 °C for 5 min. PCR products were purified using Agencourt AMPure XP (Beckman Coulter, Beverly, USA), and the concentration of the PCR products was quantified using the Qubit 2.0 Fluorometer (Life Technologies, Carlsbad, USA). The resulting libraries were sequenced with the MinION Mk 1B using flowcells FC-106D-R9.4.1 for 72 h (Oxford Nanopore Technologies).

Raw *fast5* reads were automatically basecalled using Oxford Nanopore default settings and converted to *fastq* files. Long 16S rDNA sequence reads (≤ 1700 bp; corresponding to the full-length *16S RNA* gene) were processed using the epi2me-labs/wf-16 s Nextflow workflow (v1.5.0) executed via the EPI2ME platform (Oxford Nanopore Technologies) in Kraken2 classifier mode (v2.1.3). The workflow used the default ncbi_16S_18S database set, which consist of a curated NCBI targeted loci 16S/18S reference collection, a corresponding pre-built Kraken2 database, an associated reference-to-taxid mapping file, and NCBI taxonomy files (accessed October 2025). The workflow additionally employs *fastcat* v0.20.0, *pysam* v0.23.0, *pandas* v2.2.3, *samtools* v1.21 and *TaxonKit* v0.19.0 for read processing, file handling and taxonomy annotation as part of its standard implementation.

Samples with fewer than 100 000 total reads were excluded from further analysis, resulting in 106 retained samples with per-sample reads count ranging from 122 579 to 959 294. Technical replicates originating from the same field plot were merged at the count table level, yielding 40 final composite samples with total read counts between 380 005 and 2 131 085. After denoising and prevalence filtering, taxa representing < 0.01% of total relative abundance and occurring in ≤ 5% of samples were removed prior to downstream statistical analyses.

To control potential contamination during DNA isolation, 75 μl of ZymoBIOMICS™ Microbial Community Standard II (Log Distribution) (Zymo Research) was processed in parallel with the experimental samples using the same extraction protocol. Additionally, a kit-only control (blank extraction control, ‘kitom’) was included by performing DNA extraction on an unused DNA extraction kit to detect possible reagent-derived contaminants. A sequencing process control was included, in which the library preparation was performed using nuclease-free water instead of DNA template, to identify potential contaminants introduced during library preparation and sequencing. Two reference standards were used to assess the performance of microbiomics workflow, (i)ZymoBIOMICS™ Microbial Community Standard II (Log Distribution), containing cells of ten microbial species mixed in a log-distributed abundance, and (ii) ZymoBIOMICS™ Microbial Community DNA Standard II (Log Distribution), containing a mixture of genomic DNA from the microorganisms in Microbial Community Standard II. Based on the evaluation of positive controls and known limitations of full-length 16S rDNA classification, taxonomic assignments were interpreted at the genus level and above, and species-level calls were not considered.

The Oxford Nanopore sequencing datasets supporting this study have been deposited in the NCBI database under Sequence Read Archive (SRA) numbers SRR28720296-SRR28720405, BioSample accessions SAMN40961283-SAMN40961402, and BioProject number PRJNA1099936 (https://www.ncbi.nlm.nih.gov/sra/PRJNA1099936).

### Alpha and beta-diversity of the bacterial soil community

Statistical comparisons of alpha-diversity indices (Shannon, Simpson, Richness, and Evenness) among forest types were performed in R v4.3.1^63^ using the packages *vegan* v2.6–10^62^, *ggplot2* v.4.0.0, and *ggpubr* v0.6.2^69^. Depending on data distribution, one-way ANOVA or Kruskal–Wallis tests were applied, followed by Tukey’s HSD or Dunn’s post-hoc tests, respectively, with *p-*value adjusted using the Benjamini-Hochberg (BH) correction. To standardize sequencing depth among samples and minimize bias caused by uneven read counts, the feature table was rarefied to 378 782 reads per sample, corresponding to the lowest sequencing depth.

Beta-diversity was assessed using principal coordinates analysis (PCoA) and non-metric multidimensional scaling (NMDS) to visualize differences in bacterial community among forest types. Ordinations were based on Bray–Curtis dissimilarities (genus-level relative abundances) and Jaccard dissimilarities (presence/absence data). Analyses were conducted on non0rarefied count data to retain quantitative information on relative community structure. All analyses were performed using the *vegan* and *ape* v5.8–1 packages and visualized with *ggplot2.* Differences in community composition were tested using permutational multivariate analysis of variance (PERMANOVA; 9999 permutations). To assess the homogeneity of within-group dispersions, the *betadisper* function with 9999 permutations was applied. Significant PERMDISP results (*p* < 0.05) were interpreted as evidence that differences among forest types might partly reflect dispersion effects. When global PERMANOVA indicated significant differences, pairwise comparisons were performed using *adonis2* with BH correction for multiple testing.

Relative abundance of bacterial phyla was used to calculate ecological indices commonly applied as bioindicators of soil nutrient status and trophic conditions, namely the Bacillota/Bacteroidota (F/B) ratio, the Pseudomonadota/Acidobacteriota (P/A) ratio, and the copiotroph/oligotroph index, defined as (Pseudomonadota + Bacteroidota)/(Acidobacteriota + Verrucomicrobiota). For each ratio, differences among forest types were evaluated using a non-parametric Kruskal–Wallis test, followed by pairwise Wilcoxon rank-sum tests with BH correction for multiple comparisons.

### Analysis of relationships between bacterial community composition and Biolog EcoPlate™ substrate utilization

To assess how differences in bacterial community composition relate to functional metabolic profiles, we quantified correlations between the relative abundance of dominant bacterial genera and EcoPlate substrate utilization for each forest type. Taxonomic profiles were processed at the genus level, and relative abundances were transformed using the centered log-ratio (CLR) transformation to account for compositional constraints. EcoPlate data were standardized by Z-score transformation. Spearman rank correlations were calculated separately for each forest type as well as for the full dataset. For each genus-substrate pair, the correlation coefficient (ρ) and its significance were computed using only samples belonging to the given forest type, provided that at least four non-zero and variable observations were available. Resulting *p-*values were adjusted for multiple testing using the Benjamini-Hochberg (BH) procedure. All analyses and visualizations were conducted in R using the packages *tidyverse, compositions, ggplot2, ggrepel,* and *patchwork*. Bubble-plot heatmaps were generated in two forms: (i) a combined plot aggregating correlations across all forest types, and (ii) forest-specific plots showing correlation patterns within each habitat.

### Effect of soil physicochemical properties on bacterial community structure revealed by dbRDA

To evaluate how soil physicochemical properties shape bacterial communities across different forest types, we performed distance-based redundancy analysis (dbRDA) using the *vegan* package in the R environment^62,63^. Before the analysis, the genus-level community matrix was Hellinger-transformed to reduce the influence of highly dominant taxa and to make the data more suitable for linear ordination. Environmental variables (pH, soil moisture content (SMC), soil organic matter (SOM), ash content, total carbon (SOC), nitrogen (TN), phosphorus (TP), as well as C:P, C:N, and N:P ratios) were standardized using Z-score transformation to place all variables on a comparable scale. To reduce multicollinearity, we applied a two-step filtering procedure. First, pairwise Spearman correlations were calculated, and one variable from each pair showing |ρ|> 0.7 was removed. Second, multicollinearity among the non-correlated environmental variables was assessed using variance inflation factors (VIF) computed with the function *vif.cca.* All retained variables (pH, SMC, N:P, and C:P ratios) showed VIF scores < 5, indicating low collinearity; therefore, no further exclusion was required. This procedure resulted in a reduced set of environmental variables used as constraints in dbRDA. The final dbRDA model was fitted using Bray–Curtis dissimilarity as the distance measure. Statistical significance of the overall model and individual contrained axes was assessed with 999 permutation tests. Adjusted R^2^ values were calculated to quantify the proportion of community variation explained after correcting for the number of predictors. To identify the strongest environmental gradients and the most responsive bacterial genera, we fitted vectors of environmental variables and genus abundances onto the dbRDA ordination using *envfit* function with 999 permutations. For clarity, only genera among the top 30 most abundant taxa and significant at *p* < 0.005 were shown on the final ordination plot. Ordination figures were produced in *gglpot2,* with forest types represented by ellipses, and environmental vectors shown as directional arrows proportional to their loadings in dbRDA space.

### Understorey vegetation composition as a driver of variation in soil bacterial communities

Understorey vegetation data were converted from Londo’s decimal scale to percentage cover and square-root transformed before calculating Bray–Curtis dissimilarities (*vegadist* function). Principal coordinates analysis (PCoA) was then performed on the resulting distance matrix, and the first two ordination axes (VegPCoA1-2) were retained as synthetic gradients describing variation in understorey vegetation composition. For bacterial metataxonomic data, genus-level relative abundances were Hellinger-transformed. Distance-based redundancy analysis (dbRDA) (with the model genus_hell ~ VegPCoA1 + VegPCoA2) was used to assess the extent to which variation in bacterial community composition was explained by understorey vegetation composition (R^2^adj). The significance of the overall model and individual constrained axes was evaluated using 999 permutation tests. To identify vegetation gradients and bacterial genera most strongly associated with the dbRDA ordination, we performed vector fitting using the *envfit* function (999 permutations). For bacterial predictors, the 30 most abundant genera were considered, and the top significant vectors (*p* < 0.05) with the longest projections were retained for interpretation and plotting. All analyses were conducted in R using the *vegan, tidyverse,* and *ggplot2* packages.

Next, to evaluate how understorey vegetation composition relates to variation in soil bacterial communities, we applied fourth-corner analysis and RLQ ordination. Both analyses were performed using R with *vegan, ade4, tidyverse* and *ggraph* packages^62,63,69^.

Plant species were converted to percentage cover, Hellinger-transformed, and filtered to retain only those reaching at least 10% cover in one or more plots (R matrix). Genus-level relative abundances were filtered by retaining only those with a mean relative abundances ≥ 0.5% in three or more plots, and the filtered matrix was Hellinger-transformed (L matrix). Associations between plant species and bacterial genera were tested using the fourth-corner method (*fourthcorner,* modeltype = 6). Significance was assessed using 999 permutations, and *p*-values were corrected using the FDR procedure for both G and D statistics. Significant plant-bacteria pairs (FDR-adjusted *p* < 0.05) were extracted and visualized as a bipartite interaction network.

RLQ analysis was used to summarize the joint structure between vegetation (R), bacterial composition (L), and identity-coded bacterial genera (Q). The L matrix was analyzed using correspondence analysis (*dudi.coa*), the R matrix was analyzed by PCA (*dudi.pca*) using L-derived row weights, while Q matrix was an identity matrix (each genus treated as a unique trait) analyzed by PCA using L-derived column weights. RLQ ordination (*rlq,* nf = 2) integrated the three components into a joint space. RLQ site scores were plotted by forest type with 95% confidence ellipses. To aid interpretation, the ten bacterial genera and ten plant species with strongest loadings (largest vector lengths on RLQ1-2) were displayed. To evaluate whether the observed co-structure between vegetation and bacterial communities exceeded that expected by chance, we performed a global permutation test using (*randtest*.*rlq*, 999 permutations).

### Integrated block sPLS analysis of bacteria, understorey vegetation and soil properties

To integrate variation in bacterial community composition, understorey vegetation and soil properties, we used sparse multiblock partial least squares (block.sPLS) as implemented in the *mixOmics* R package. Three data blocks were constructed for the 40 plots: (i) Bacteria, comprising genus-level relative abundances Hellinger-transformed as in the ordination analyses, (ii) Plants, comprising Hellinger-transformed percent cover of understorey species, and (iii) Soil, containing standardized values of six physicochemical variables (pH, SOM, SOC, SMC, total N and total P). We first manually tuned the sparsity parameters (keepX) that control the number of variables retained on each component. For each keepX combination, a block.sPLS model with two components was fitted, and we calculated the mean absolute correlation between the latent variates of the three blocks as a performance criterion. This tuning procedure identified an optimal configuration with keepX = 15 genera and 30 plant species (mean |cor|= 0.741). However, models with 30 bacterial genera performed almost as well (mean |cor|= 0.725), indicating a very flat performance landscape. To retain a broader representation of bacterial diversity, we therefore chose a final model with 30 bacterial genera and 30 plant species per component, while keeping all soil variables.

The final block.sPLS model (ncomp = 2) was fitted in canonical mode with scaling of all predictors. To evaluate whether the multiblock structure was stronger than expected by chance, we performed a permutation test (999 permutations) in which sample identities in the plant block were randomly permuted while bacterial and soil blocks were kept fixed. The permutation p-value was calculated as the proportion of permuted models with mean |cor| greater than or equal to the observed value.

## Supplementary Information

Supplementary Information 1.

Supplementary Information 2.

## Data Availability

The data used to support the findings of this study can be made available by the corresponding author upon request.
