# Supplementary material for: Bacteria-soil–plant linkages underlie the mosaic structure of the soil bacterial communities in near-natural stands of Białowieża Primeval Forest
Source: Sci Rep. 2026 Mar 13;16:13444. doi: 10.1038/s41598-026-40694-1 (PMC13111676; doi:10.1038/s41598-026-40694-1)
Supplement: Supplementary file 1 — Supplementary Information 1. [file 41598_2026_40694_MOESM1_ESM.pdf]

Supplementary Figures

Bacteria-soil-plant linkages underlie the mosaic structure of the soil bacterial communities in near-natural stands of Białowieża Primeval Forest

Justyna M. Drewnowska[1,2,*], Wioleta Lewandowska[1,3], Piotr Zieliński[4], Piotr Jadwiszczak[5], Bogdan Jaroszewicz[6], Andrzej Keczyński[7], Olga Hummel[8], Piotr Majewski[9], Izabela Święcicka[1,2]

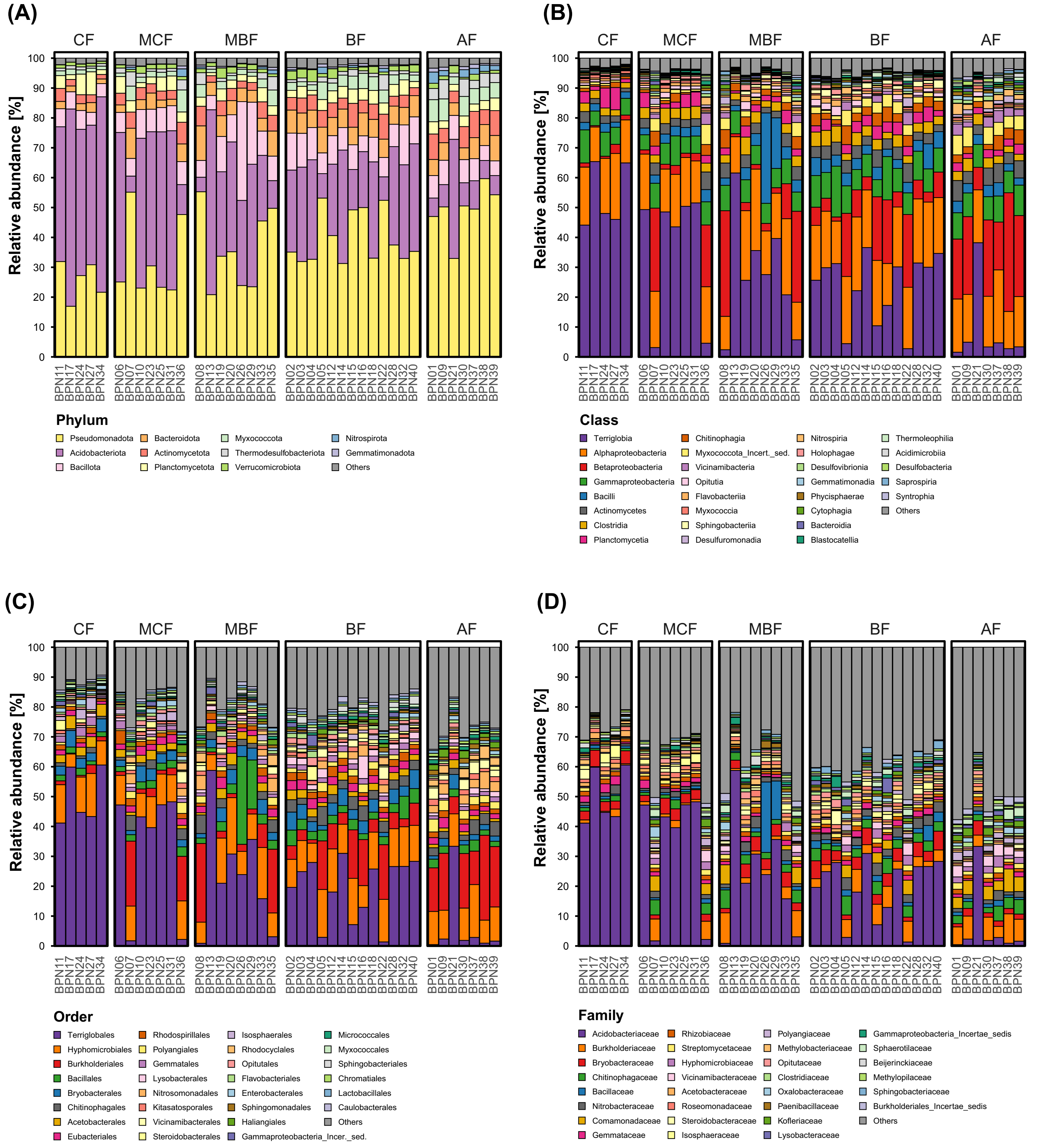

Figure S1. Relative abundance of dominant bacterial taxa at different taxonomic levels across forest types.

Stacked bar charts showing the relative abundance of the most abundant bacterial taxa (Top30) at the (A) phylum, (B) class, (C) order, and (D) family levels in topsoil samples from coniferous (CF), mixed coniferous, (MCF), mixed broadleaf (MBF), broadleaf (BF), and alder forests (AF). Only taxa representing ≥ 1% of the total abundance in at least one sample were retained; all remaining taxa were grouped as *Others* (grey).

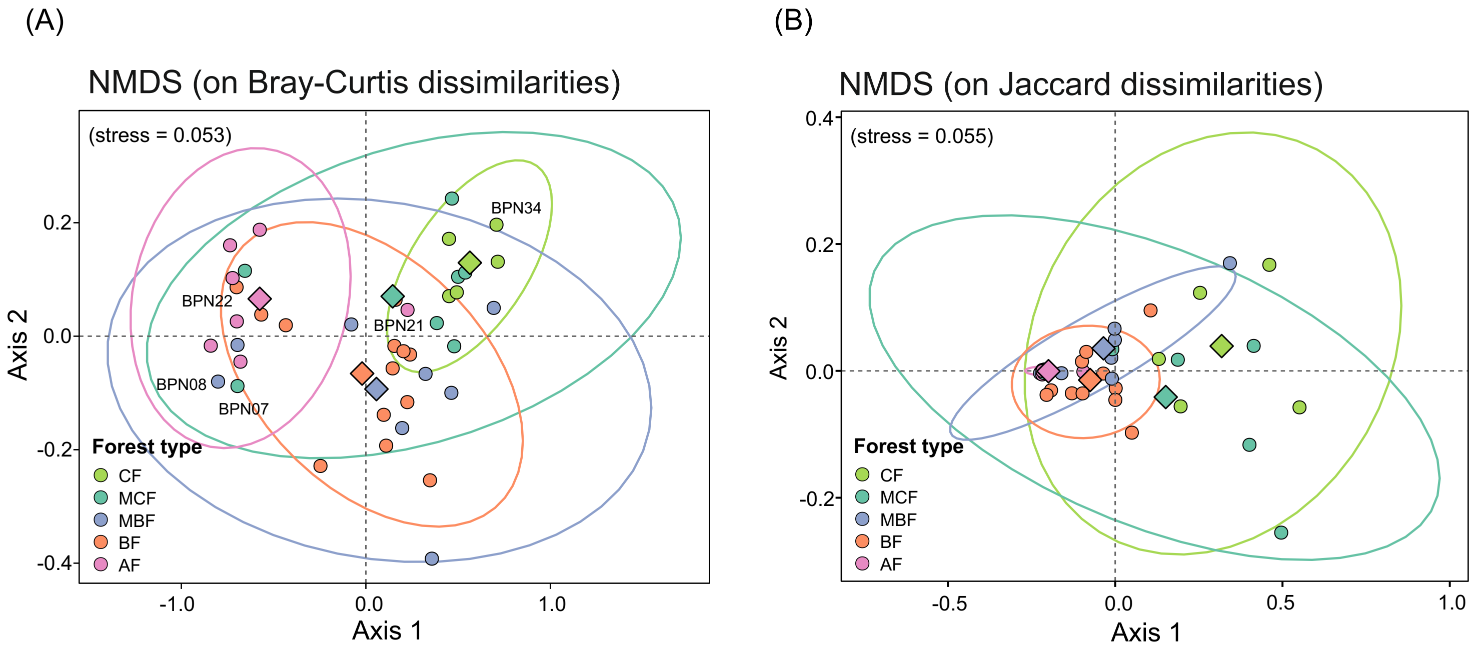

Figure S2. Non-metric multidimensional scaling (NMDS) ordination of bacterial community β-diversity at the genus level.

NMDS ordination based on (A) Bray-Curtis and (B) Jaccard (presence/absence) dissimilarities illustrates patterns of compositional differences among forest types (CF, coniferous forest; MCF, mixed coniferous forest; MBF, mixed broadleaf forest; BF, broadleaf forest; and AF, alder forest). Ellipses denote 95% confidence intervals for each forest types and diamonds indicate group centroids (mean coordinates). Sample labels mark statistical outliers identified as points lying beyond the 95% confidence ellipse for their respective forest type.

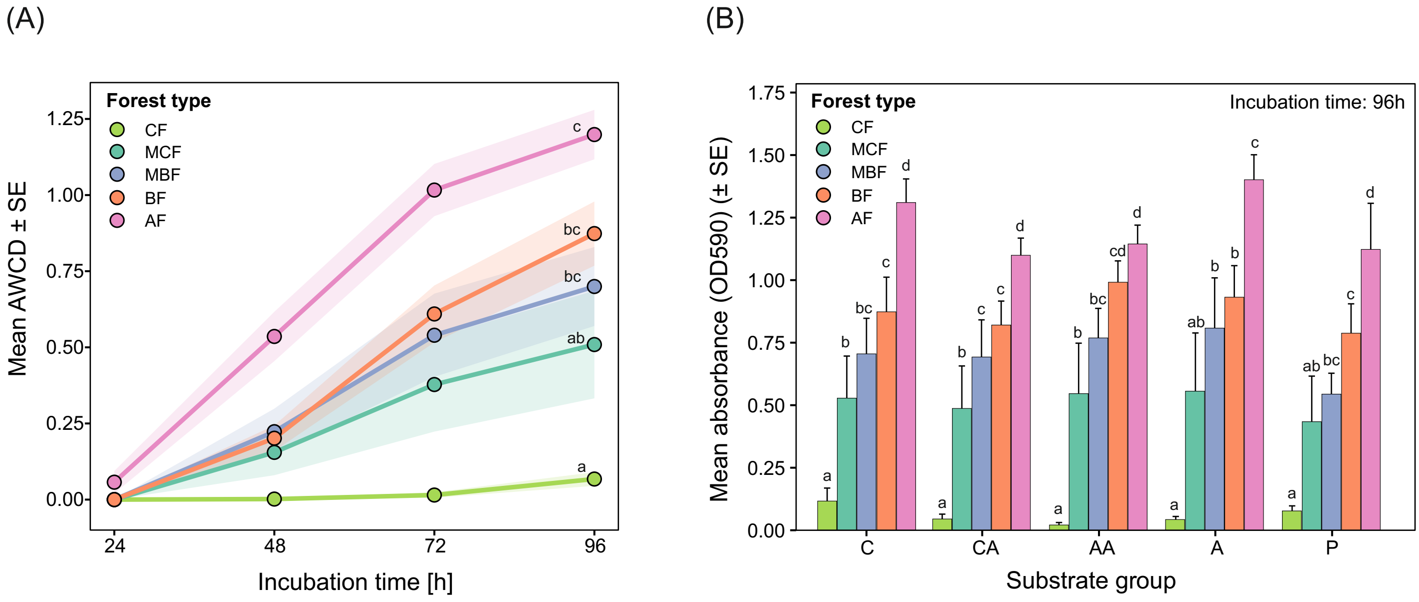

Figure S3. Dynamics and utilization patterns of carbon substrates in soil microbial communities from Białowieża National Park (based on the Biolog EcoPlate assay)

(A) Temporal changes in average well-color development (AWCD) across forest types during incubation. (B) Mean utilization of five major substrate groups after 96 h of incubation. Significant differences among forest types, based on Kruskall-Wallis tests followed by pairwise Wilcoxon comparisons with BH correction ($p<0.05$), are indicated by lowercase letters above the bars.

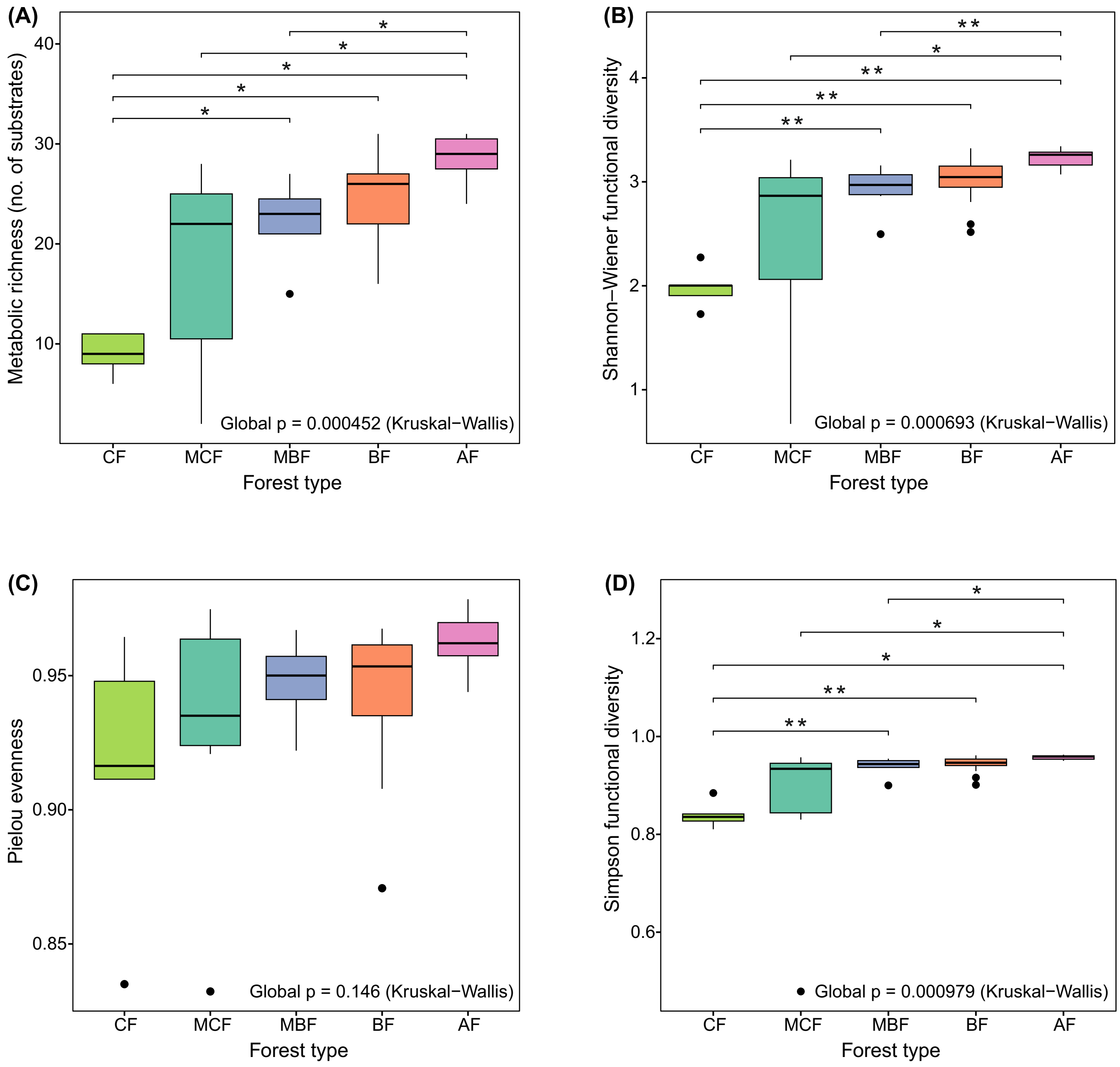

Figure S4. Functional alpha-diversity indices of microbial activity based on Biolog EcoPlate assay.

Functional alpha-diversity indices: (A) metabolic richness (number of utilized substrates), (B) Shannon-Wiener functional diversity, (C) evenness, (D) Simpson diversity across five forest types (CF, coniferous; MCF, mixed coniferous forest; MBF, mixed broadleaf forest; BF, broadleaf forest; and AF, alder forest). Boxplots show the median (horizontal line), interquartile range (box), and outliers (individual points). Global Kruskal-Wallis *p*-values are shown on each panel. Brackets and asterisks indicate significant pairwise differences between forest types (BH-adjusted p<0.05*, <0.01**, <0.001***).

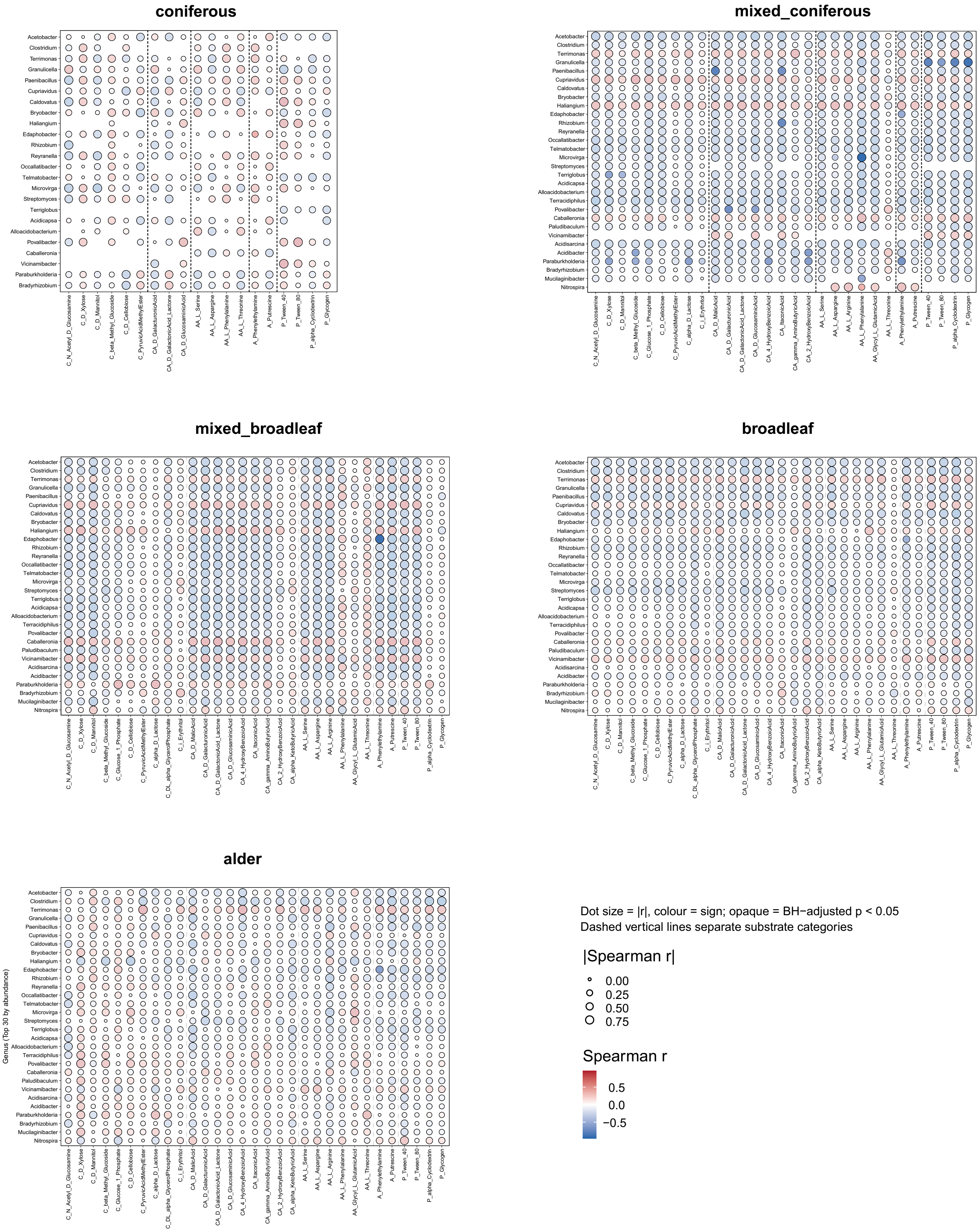

Figure S6. Correlation between bacterial genera and Biolog EcoPlate substrate utization profiles within different forest types.

Spearman correlation between relative abundances of the 30 most abundant bacterial genera EcoPlate substrate utilization profiles within different forest types. Circle color indicates the direction of the correlation, while cirle size is proportional to |ρ|.

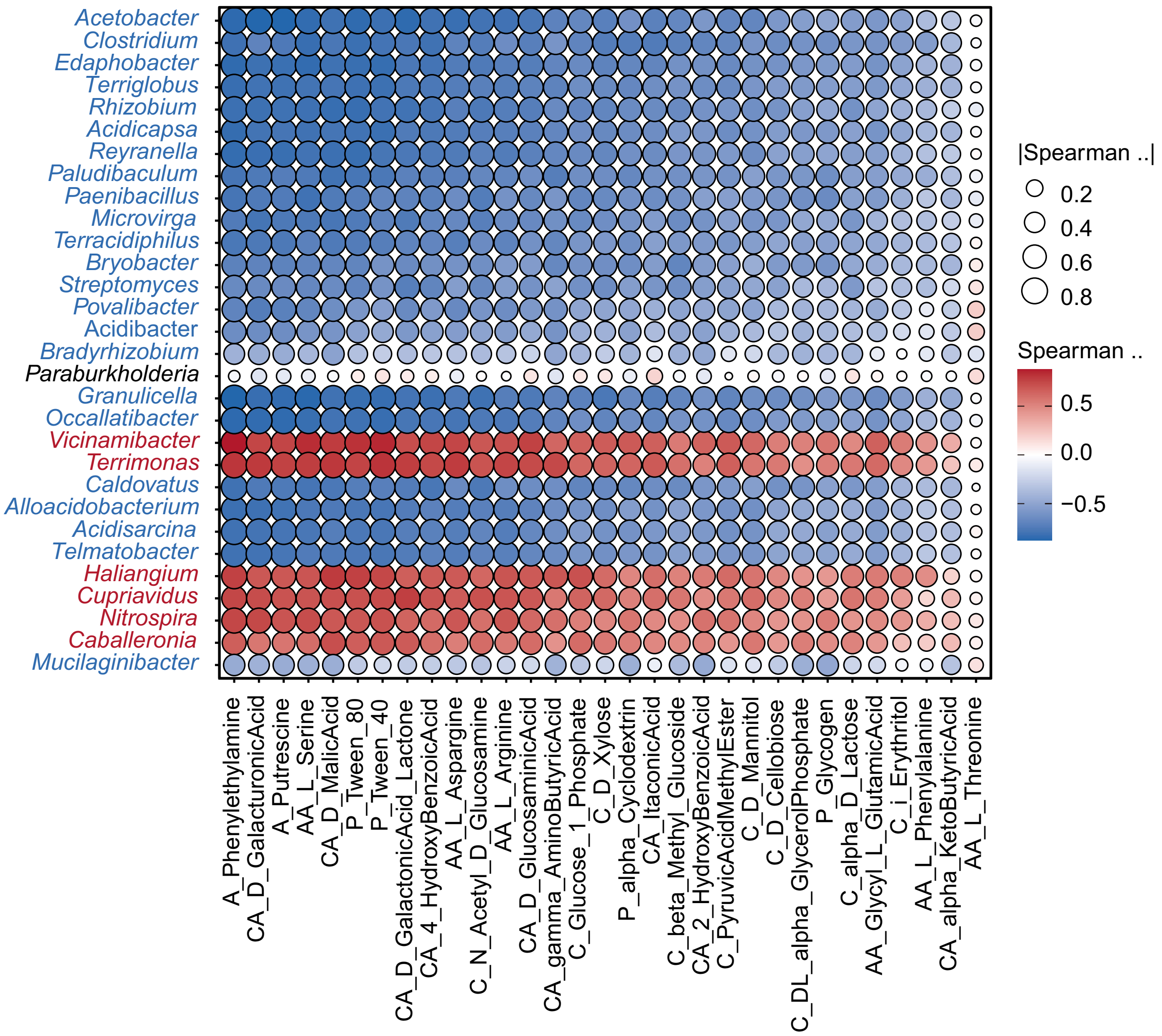

Figure S6. Correlation between bacterial genera and Biolog EcoPlate substrate utization profiles.

Spearman correlation between relative abundances of the 30 most abundant bacterial genera EcoPlate substrate utilization profiles across all forest types (combined). Circle color indicates the direction of the correlation, while cirle size is proportional to |ρ|.

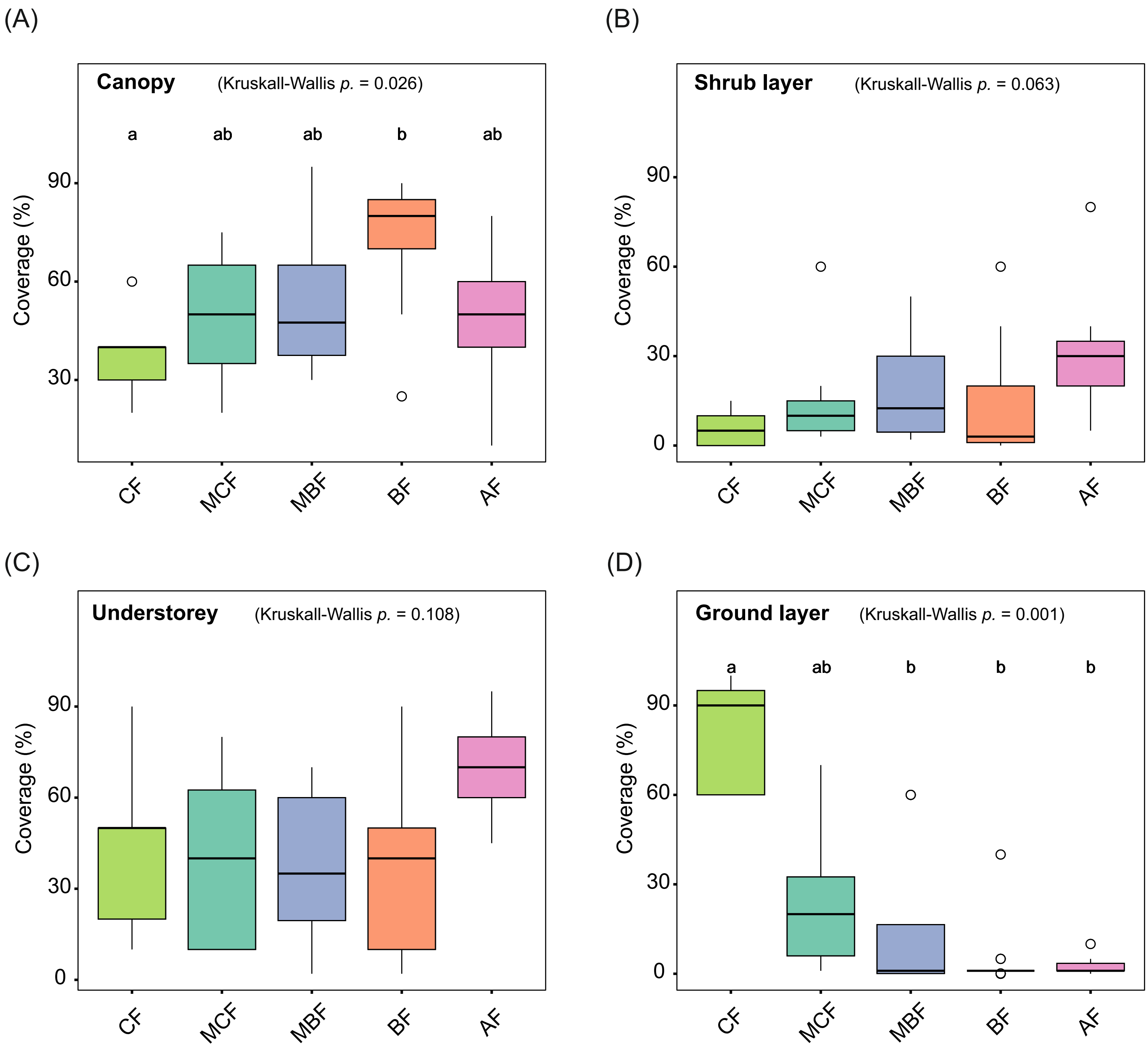

Figure S7. Vegetation cover of four vertical layers across forest types in Białowieża National Park.

Boxplots show percentage cover for (A) canopy, (B) shrub, (C) understorey, and (D) ground layers. Boxes represent interquartile ranges, central lines denote medians, and points indicate outliers. Forest types: CF, coniferous forest; MCF, mixed coniferous forest; MBF, mixed broadleaf forest; BF, broadleaf forest; AF, alder forest.

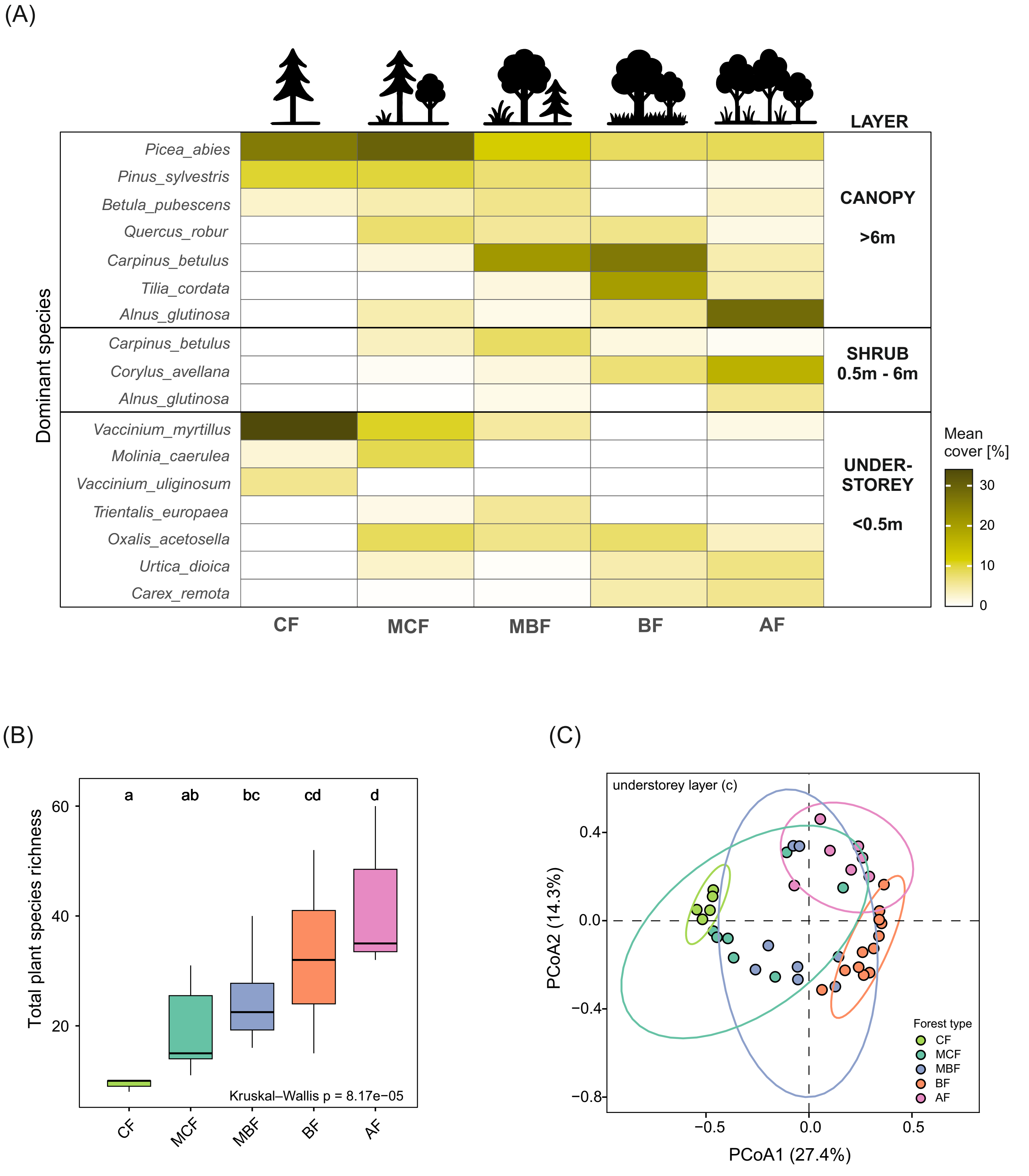

Figure S8. Vegetation structure, species richness, and understorey community composition across five forest types in Białowieża National Park.

(A) Vertical structure of dominant plant species across forest types. Heatmap shows dominant taxa in the canopy, shrub and undersotrey layers. Dominance was defined as ≥ 5% constancy and ≥ 20% mean cover based on vegetation surveys conducted in July 2019 on 40 plots (10 x 10 m.). (B) Total plant species richness calculated as the total number of vascular plant species across the three vegetation layers. Statistical differences among forest types were tested using the Kruskal-Wallis test followed by post-hoc pairwise comparisons with BH correction. (C) Principal coordinates analysis (PCoA) of understorey community composition based on Bray-Curtis dissimilarities.PERMANOVA (adonis2, 999 permutations) was used to test differences among forest types, and homogeneity of multivariate dispersion was evaluated with betadisper. Ellipses represent 95% confidence intervals. Forest types: AF, alder forest; BF, broadleaf forest; MBF, mixed broadleaf forest; MCF, mixed coniferous forest; CF, coniferous forest.

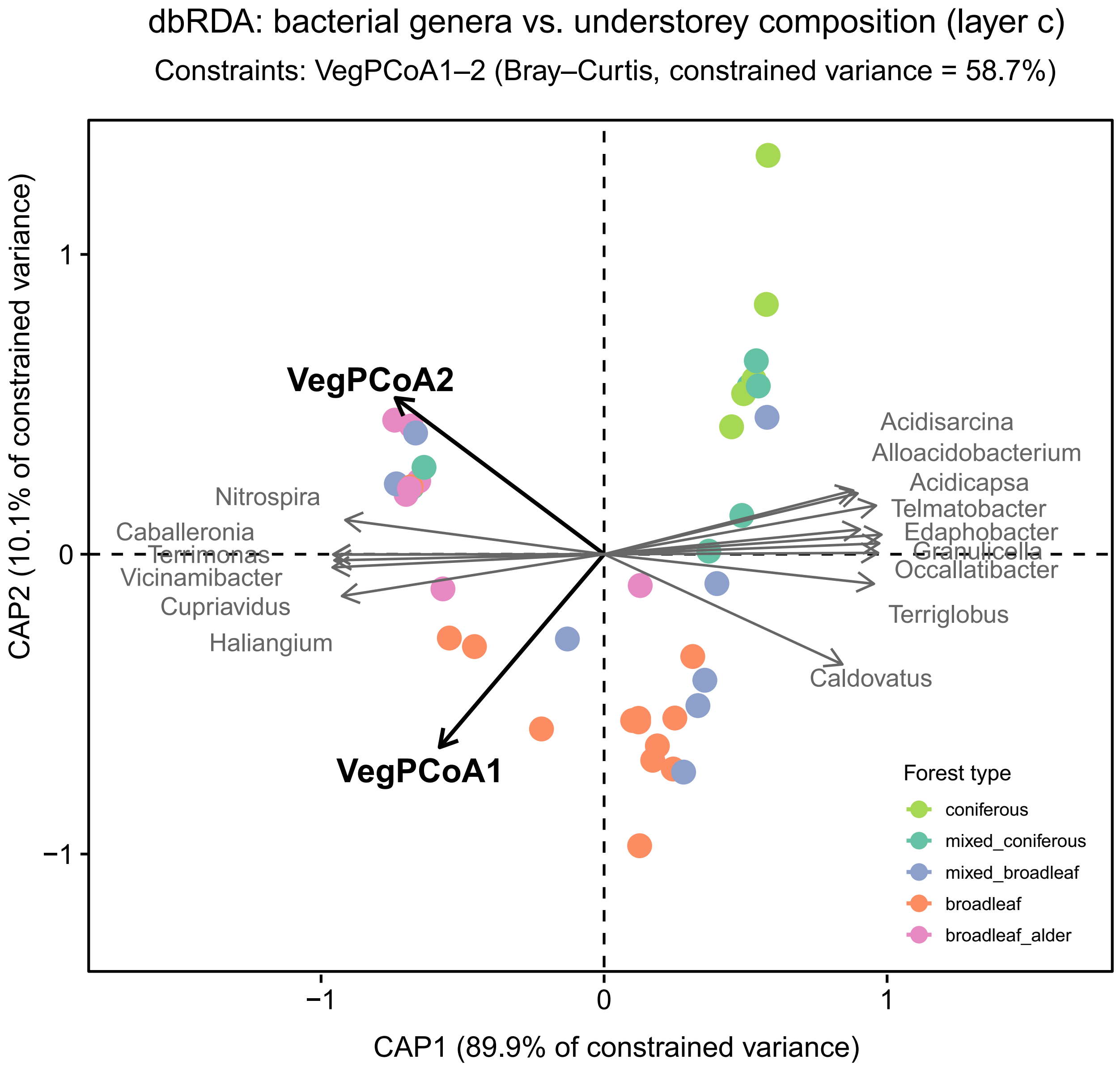

Figure S9. Distance-based redundancy analysis (dbRDA) showing the relationship between understorey vegetation composition and soil bacterial community structure across five forest types.

dbRDA ordination of bacterial community composition constrained by the first two PCoA axes of understorey vegetation (VegPCoA1-A2). The two vegetation gradients explained 41.7% of variation in plant understorey composition (VegPCoA1 = 27.4%, VegPCoA2 = 14.3%). The dbRDA model was highly significant (*p* = 0.001), with vegetation explaining 58.7% of the variation in bacterial communities (adjusted R2 = 0.564). Black vectors indicate fitted vegetation gradients (envifit, *p. = 0.001*), and grey vectors represent significant bacterial genera *p<0.05*).

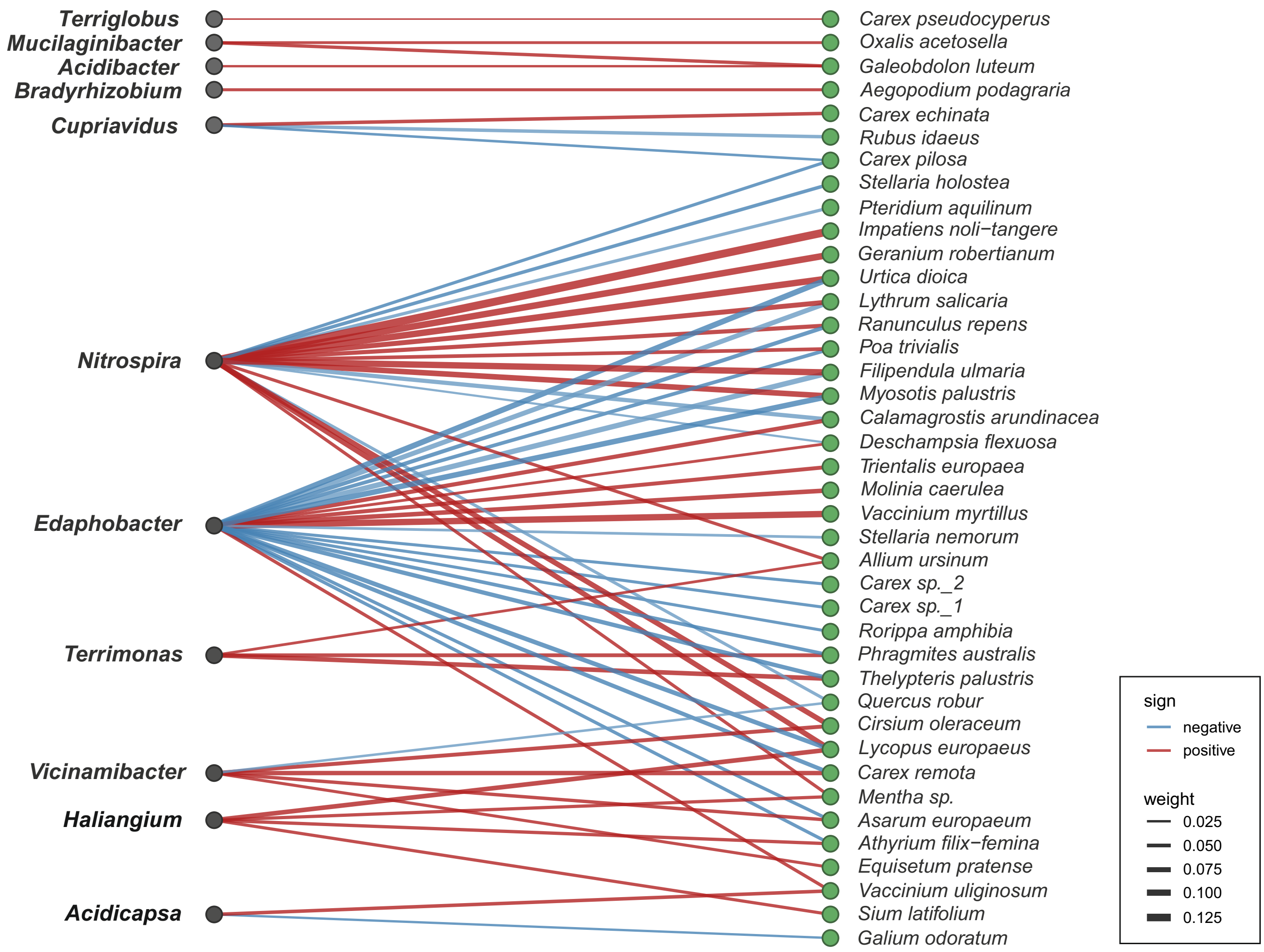

Figure S10. Bipartite network of significant plant-bacteria associations identified by the fourth-corner analysis.

Bipartite network visualising all significant associations between undersorey plant species and bacterial genera detected by the fourth-corner analysis (FDR-adjusted p. <0.05; n = 62 significant links). Edge thickness is proportional to the absolute correlation coefficient ([r|), and edge colour indicates the direction of association (red = positive, blue = negative).
